# Adult bone marrow progenitors become decidual cells and contribute to embryo implantation and pregnancy

**DOI:** 10.1371/journal.pbio.3000421

**Published:** 2019-09-12

**Authors:** Reshef Tal, Shafiq Shaikh, Pallavi Pallavi, Aya Tal, Francesc López-Giráldez, Fang Lyu, Yuan-Yuan Fang, Shruti Chinchanikar, Ying Liu, Harvey J. Kliman, Myles Alderman, Nicola Pluchino, Jehanzeb Kayani, Ramanaiah Mamillapalli, Diane S. Krause, Hugh S. Taylor

**Affiliations:** 1 Department of Obstetrics, Gynecology and Reproductive Sciences, Yale School of Medicine, New Haven, Connecticut, United States of America; 2 Yale Center for Genome Analysis (YCGA), Yale University, New Haven, Connecticut, United States of America; 3 Department of Laboratory Medicine, Yale School of Medicine, New Haven, Connecticut, United States of America; B.C. Cancer Agency, CANADA

## Abstract

Decidua is a transient uterine tissue shared by mammals with hemochorial placenta and is essential for pregnancy. The decidua is infiltrated by many immune cells promoting pregnancy. Adult bone marrow (BM)-derived cells (BMDCs) differentiate into rare populations of nonhematopoietic endometrial cells in the uterus. However, whether adult BMDCs become nonhematopoietic decidual cells and contribute functionally to pregnancy is unknown. Here, we show that pregnancy mobilizes mesenchymal stem cells (MSCs) to the circulation and that pregnancy induces considerable adult BMDCs recruitment to decidua, where some differentiate into nonhematopoietic prolactin-expressing decidual cells. To explore the functional importance of nonhematopoietic BMDCs to pregnancy, we used Homeobox a11 (Hoxa11)-deficient mice, having endometrial stromal-specific defects precluding decidualization and successful pregnancy. Hoxa11 expression in BM is restricted to nonhematopoietic cells. BM transplant (BMT) from wild-type (WT) to Hoxa11^−/−^ mice results in stromal expansion, gland formation, and marked decidualization otherwise absent in Hoxa11^−/−^ mice. Moreover, in Hoxa11^+/−^ mice, which have increased pregnancy losses, BMT from WT donors leads to normalized uterine expression of numerous decidualization-related genes and rescue of pregnancy loss. Collectively, these findings reveal that adult BMDCs have a previously unrecognized nonhematopoietic physiologic contribution to decidual stroma, thereby playing important roles in decidualization and pregnancy.

## Abstract 2

Adult bone marrow–derived progenitor cells make a nonhematopoietic contribution to maternal decidua by differentiating into decidual stromal cells during mouse pregnancy. Their ability to rescue pregnancy loss in mice with decidual stromal cell-specific deficiency shows that these bone marrow–derived mesenchymal progenitors play an important role in establishing and maintaining pregnancy.

## Introduction

The decidua is a transient tissue lining the uterus of mammals with hemochorial placenta (mice, humans, and numerous other mammalian species) and is essential for pregnancy in these species. In humans, decidualization, the transformation of endometrial stromal cells into epitheloid decidual cells, occurs throughout the endometrium at the end of the implantation window (approximately 10 days after ovulation) independently of pregnancy. This differentiation process is dependent on 3′,5′-cyclic AMP (cAMP) and progesterone signaling pathways that lead to profound transcriptome and proteome changes [1]. The differentiated decidua exhibits only a short period of receptivity known as “the window of implantation,” when embryo attachment and implantation is possible [2]. In the absence of blastocyst implantation, progesterone levels decline and the decidualized endometrium undergoes timely destruction, leading to menstruation. With blastocyst implantation, the decidua grows and is maintained throughout gestation [1]. In mice, endometrial decidualization occurs later, initiated by blastocyst attachment to the uterine epithelium on embryonic day 4.5 (E4.5) and is localized to the implantation sites [3]. Although embryo quality is an important determinant of implantation, temporally coordinated differentiation of endometrial stromal cells into decidual cells to attain uterine receptivity, and a synchronized dialog between maternal and embryonic tissues are crucial for successful implantation [2]. These specialized decidual cells play key roles in nutritional sensing, endocrine regulation, immune tolerance, and evaluation of embryo quality [4,5].

It is well known that uterine implantation sites are the site of infiltration of many peripherally derived immune cells [6], including natural killer (NK) cells, macrophages, and T cells, which play important roles at the maternal–fetal interface to promote successful pregnancy [7–10]. While it is well established that many decidual immune cells originate in the bone marrow (BM) [7,11], it remains unknown whether adult BM-derived cells (BMDCs) can give rise to nonhematopoietic cells in the decidua. Kearns and Lala have previously proposed that BMDCs may also give rise to stromal decidual cells in pregnancy based on reconstitution of BM cells in the fetal period [12,13]. However, others could not find such evidence [14], and this concept was further challenged by investigators demonstrating that resident endometrial stromal cells differentiate into decidual cells [15]. Moreover, because BM reconstitution was performed during fetal life, it remained unknown whether BMDCs migrating to the uterus during adult life can give rise to nonhematopoietic decidual cells. Adult BMDCs have been shown to travel in the circulation and contribute to tissue repair and regeneration of various organs [16]. In the uterus, adult BMDCs have been detected in both human [17–19] and mouse [18,20–23] endometrium, and demonstrated to give rise to various nonhematopoietic endometrial cells, including epithelial, stromal, and endothelial cells (ECs) in the nonpregnant state, suggesting that BMDCs may serve as a source of progenitor cells for endometrial regeneration. However, whether adult BMDCs give rise to nonhematopoietic decidual cells, their temporal and spatial nonhematopoietic contribution to implantation and pregnancy, and whether implantation is a stimulus for the migration of these progenitors to the uterus remain uncharacterized. Moreover, the functional importance of such cells to embryo implantation and pregnancy development is unknown.

In this study, we utilized our previously described non-gonadotoxic BM transplant (BMT) regimen [24] to investigate the nonhematopoietic physiologic contribution of adult BMDCs to decidual stroma during pregnancy in wild-type (WT) mice. In addition, to explore the functional importance of BMDCs to implantation and pregnancy, we have used Homeobox a11 (Hoxa11) genetic knockout (KO) mice models, which are associated with endometrial stromal defects leading to decidualization failure and lack of pregnancy in homozygous (^−/−^) and pregnancy loss in heterozygous (^+/−^) mice. We chose the Hoxa11 KO model because Hoxa11 has been shown to be expressed in the BM exclusively in nonhematopoietic mesenchymal stem/stromal cell populations [25], allowing us to dissect the potential nonhematopoietic BMDCs functional contribution to pregnancy. Our data show that embryo implantation and pregnancy are associated with an increase in mobilization of mesenchymal stem cells (MSCs) to the circulation, and recruitment of BMDCs to the uterus, where BMDCs are a source of functional nonhematopoietic decidual cells. In addition, transplantation of BM from Hoxa11-expressing WT donors leads to endometrial stromal expansion, gland formation, and decidualization in Hoxa11^−/−^ mice. Moreover, BMT from WT donors results in normalization of uterine expression of numerous decidualization-related genes, leading to rescue of pregnancy losses in Hoxa11^+/−^ mice. Collectively, these data highlight the important nonhematopoietic role that adult BMDCs play in implantation and pregnancy maintenance.

## Results

### Pregnancy is a strong stimulus for BMDCs recruitment to the uterus

**Fig 1:**
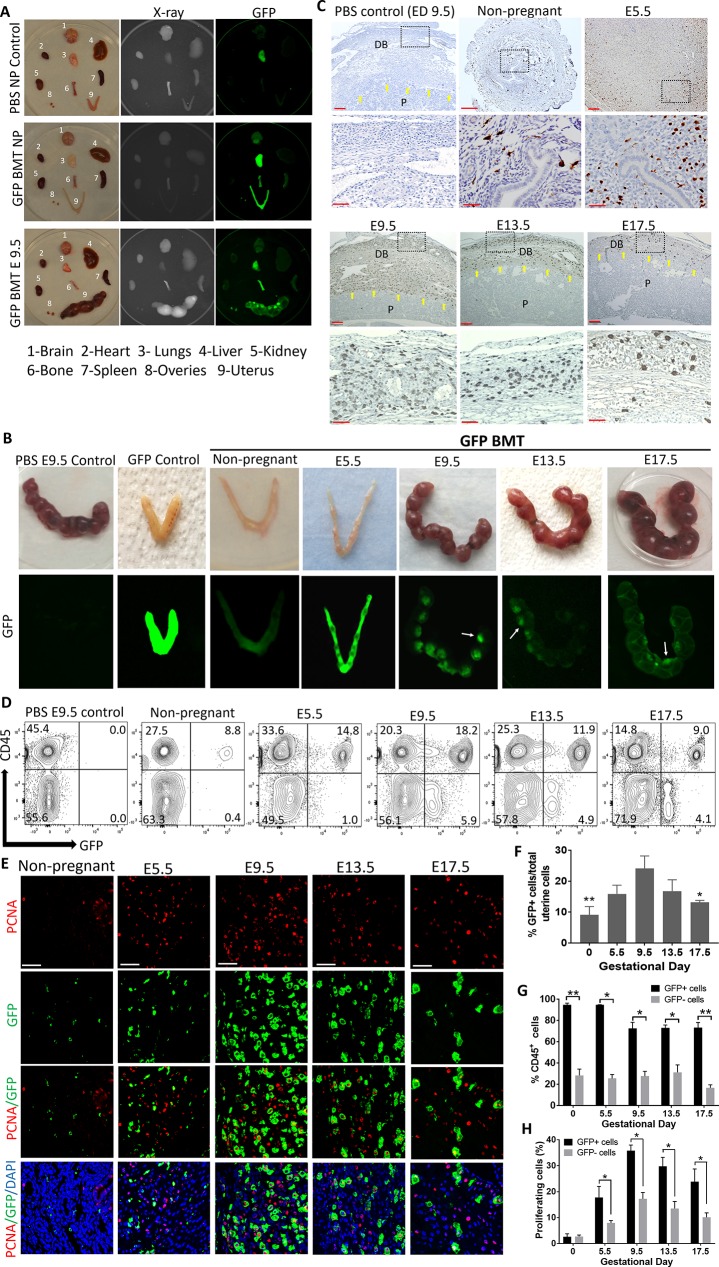
Spatial and temporal contribution of BMDCs to decidua throughout mouse pregnancy. (A) Biodistribution of BM-derived (GFP^+^) cells showing preferential recruitment to the pregnant uterus as compared with other body organs. Top panel, PBS nonpregnant control; middle panel, nonpregnant BMT; bottom panel, BMT at E9.5. (B) Engraftment of BMDCs (green) in uteri in nonpregnant state and throughout pregnancy in WT mice receiving BMT from GFP donors. PBS-injected pregnant mouse (E9.5) is shown as negative control, and GFP transgenic mouse is shown as positive control. White arrows indicate the preferential localization of BMDCs to the implantation site. (C) Uterine tissue sections of pregnant mice stained with anti-GFP antibody (brown) showing the localization of BMDCs in decidua during the course of pregnancy. Yellow arrows point to the maternal–fetal interface demarcating the maternal decidua basalis (DB) from fetal placenta (P). Scale bars, 100 μm (top panel) and 50 μm (bottom panel). (D) Representative graphs of flow cytometry of uterine cells demonstrating the temporal changes in hematopoietic (CD45^+^) and nonhematopoietic (CD45^−^) GFP^+^ BMDCs populations during the course of pregnancy. Numbers in each quadrant indicate percentage of cells. (E) Immunofluorescence of uterine tissue sections showing colocalization of PCNA-positive proliferating cells (red) and GFP-positive BMDCs (green) during the course of pregnancy; sections were counterstained with DAPI (blue). Scale bar, 50 μm. (F) Summary of flow cytometry analysis of percentage of GFP^+^ cells in the uterus during pregnancy (*n* = 5–7). **p ≤ 0.01 versus E9.5, E13.5, and E17.5. *p ≤ 0.05 versus E9.5. (G) Summary of flow cytometry analysis of percentage of GFP^+^ and GFP^−^ cells in the uterus that are either CD45^+^ or CD45^−^ during the course of pregnancy (*n* = 5–7). *p < 0.01, **p < 0.001. (H) Quantification of proliferating PCNA^+^GFP^+^ BMDCs in the uterus during the course of pregnancy (*n* = 4–7). All bar graphs are mean ± SEM. *p ≤ 0.05. See also S1 Fig. Underlying data are available in S1 Data. BM, bone marrow; BMDC, BM-derived cell; BMT, BM transplant; DB, decidua basalis; E, embryonic day; GFP, green fluorescent protein; NP, nonpregnant; P, fetal placenta; PCNA, proliferating cell nuclear antigen; WT, wild-type.

To explore the contribution of adult BMDCs to the uterus during implantation and pregnancy development, we utilized our previously described non-gonadotoxic BMT model [24] (S1A Fig). WT female mice underwent submyeloablation using a regimen consisting of 5-fluorouracil (5-FU) and stem cell factor followed by BMT from green fluorescent protein (GFP)-expressing syngeneic donors. Successful BM engraftment was confirmed by flow cytometry analysis of peripheral blood on day 21 post-BMT, demonstrating approximately 45% donor chimerism (S2A and S3A Figs), consistent with our previous experience using this model [24]. Our first objective was to characterize the spatial and temporal distribution of BMDCs in the uterus during pregnancy. A pregnancy time course experiment was performed, and mice were killed at various gestational time points. Biodistribution analysis of GFP signal showed that BM-derived GFP^+^ cells migrated preferentially to the uterus during pregnancy compared with other organs (Fig 1A). The lung was the only organ that showed some nonspecific autofluorescence, as seen in the phosphate-buffered saline (PBS)-injected control. Moreover, visualization of the intact uterus showed GFP signal in pregnant uteri, with signal enhancement at sites of implantation (Fig 1B). Dissection of the pregnant uterus demonstrated that the GFP^+^ cells localized to the maternal side of the uterus and were not arising from the placenta or embryo (S1B Fig). Histological analysis of implantation site sections by GFP immunostaining confirmed these findings, revealing that BM-derived GFP^+^ cells were concentrated specifically in the implantation site on the maternal side, reaching a peak at mid-gestation (E9.5) (Fig 1C). GFP^+^ cells were localized abundantly in the stroma of the decidua on the mesometrial side, but also on the antimesometrial side (S4 Fig). Flow cytometry analysis of uterine implantation sites following removal of the placenta/embryo demonstrated that the percentage of BM-derived GFP^+^ cells in the implantation site increased as pregnancy progressed to mid-gestation; it increased significantly on the day of implantation (E5.5) (15.8%) as compared with the nonpregnant state (9.2%), peaking on E9.5 (24.1%), followed by a gradual decrease on E13.5 (16.7%) and peripartum (E17.5) (13.1%) (Fig 1D and 1F). As the observed increase in GFP^+^ cells in the decidua during pregnancy could be due to local proliferation of BMDCs and not only to increased recruitment, we assessed cell proliferation using proliferating cell nuclear antigen (PCNA) staining. The percentage of proliferating cells in the nonpregnant uterine stroma was minimal and not different between the GFP^+^ (BM-derived) (2.6%) and resident GFP^−^ (non–BM-derived) cells (2.7%) (Fig 1E and 1H). In contrast, cell proliferation in the pregnant uterine stroma gradually increased throughout gestation, peaking on E9.5, and was significantly greater in the GFP^+^ subpopulation versus the GFP^−^ resident subpopulation at all gestational time points (Fig 1E and 1H), suggesting that the increase in the BMDC population in the decidua during pregnancy is at least partly due to preferential local proliferation of recruited BMDCs.

### Numerous uterine BMDCs are nonhematopoietic and differentiate into stromal decidual cells in pregnancy

**Fig 2:**
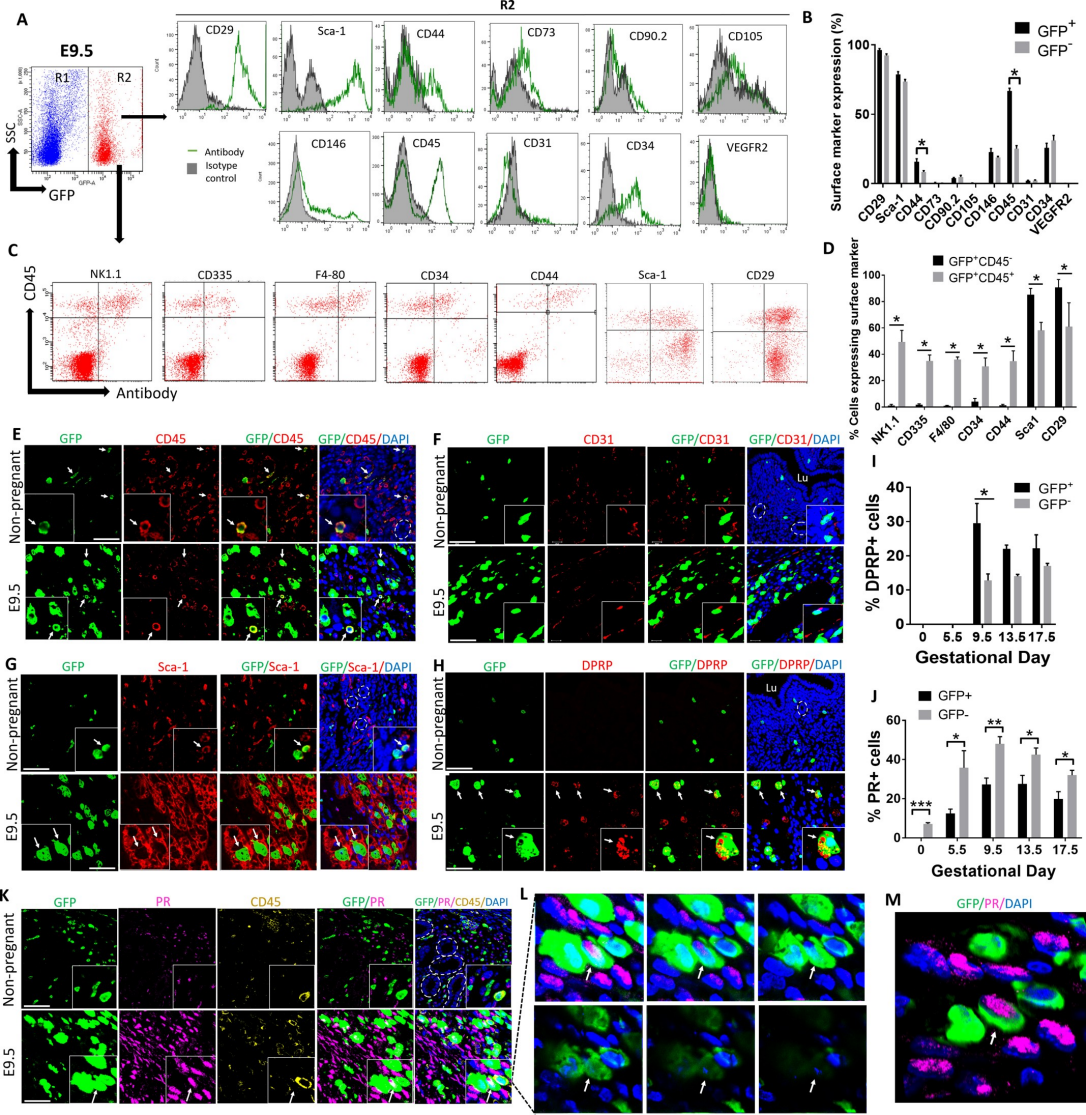
Characterization of uterine BMDCs throughout pregnancy. (A) Flow cytometry analysis of BMDCs in uterine implantation site on E9.5. Cells gated in R2 are BM derived (GFP^+^) uterine cells, while cells gated in R1 are non–BM-derived resident (GFP^−^) uterine cells. Histograms represent GFP^+^ cells (R2) from E9.5 implantation sites that are stained with the indicated antibodies (green line) and respective isotype controls (filled) (*n* = 4). (B) Quantification of percentage of BM-derived (GFP^+^) and non–BM-derived (GFP^−^) uterine cells expressing the various cell surface markers shown in (A) (*n* = 4), *p < 0.05. (C) Flow cytometry analysis of E9.5 BM-derived (GFP^+^) uterine cells (R2) using CD45 in combination with various surface marker antibodies. (D) Quantification of surface markers shown in (C), on BM-derived nonhematopoietic decidual cells (GFP^+^CD45^−^) and BM-derived hematopoietic cells (GFP^+^CD45^+^) (*n* = 4–5), *p < 0.01. (E, F, G, H, K) Immunofluorescence photomicrographs of E9.5 mesometrial decidua sections or nonpregnant mice uteri sections demonstrating co-staining of GFP^+^ BMDCs (green) with (E) CD45 (red), (F) CD31 (red), (G) Sca-1 (red), (H) DPRP (red), (K) progesterone receptor (PR) (pink), and CD45 (yellow). Sections were counterstained with DAPI showing nuclei (blue). Insets show higher magnification photomicrographs. White arrows point to BMDCs colocalizing with their respective markers. White dashes are encircling endometrial glands. Scale bars, 50 μm. (L and M) A z-stack series (L) and a 3D image (M) of the inset from (K) demonstrating a single BM-derived GFP^+^ cell co-expressing PR but negative for CD45 (white arrow). (I) Quantification of BMDCs (GFP^+^) or non-BMDCs (GFP^−^) positive for DPRP throughout gestation (*n* = 3). *p ≤ 0.01. (J) Quantification of BMDCs (GFP^+^) or non-BMDCs (GFP^−^) positive for PR throughout gestation (*n* = 3–5). *p ≤ 0.05, **p ≤ 0.01, ***p ≤ 0.001. In all panels, bar graphs represent mean ± SEM. See also S3–S5 Figs. Underlying data are available in S1 Data. BM, bone marrow; BMDC, BM-derived cell; DPRP, decidual prolactin-related protein; GFP, green fluorescent protein; PR, progesterone receptor; Sca-1, stem cell antigen-1; SSC, side scatter; VEGFR2, vascular endothelial growth factor receptor 2.

During early mouse pregnancy, there is a large influx of NK cells to the developing decidua, with NK cells comprising up to approximately 70% of all uterine immune cells at mid-gestation. It is well established that these NK cells concentrate in the decidua exclusively on the mesometrial side of the implantation site and no NK cells are found on the antimesometrial side [6]. Our histological analysis of immunosections stained for GFP revealed that BMDCs were localized abundantly in the decidua on the mesometrial side, but also on the antimesometrial side, indicating that these BMDCs were not just NK cells (S4 Fig). To characterize the phenotype of decidual BMDCs during pregnancy, we utilized flow cytometric analysis (Fig 1D). In the nonpregnant uterus, BM-derived GFP^+^ cells were mostly positive for the pan-leukocyte marker CD45 (94.8%), indicating that the majority of this population consisted of immune cells. In contrast, only a small subset of non–BM-derived resident (GFP^−^) uterine cells in the nonpregnant uterus were positive for CD45 marker (28.3%). During pregnancy, however, the proportion of CD45^+^ cells within the total GFP^+^ uterine cell population gradually decreased, reaching a nadir at mid-gestation on E9.5 (72.4%) (Fig 1D and 1G). This trend was despite a marked overall increase in the total number of GFP^+^ cells in the uterus (Fig 1F), suggesting that many BMDCs recruited to the uterus in pregnancy were progenitors that differentiated into nonhematopoietic (CD45^−^) cells. Flow cytometric analysis of implantation sites on E9.5, the time of peak concentration of BMDCs (24.1%), revealed that the BM-derived GFP^+^ uterine cells (R2) expressed the cell surface markers CD29 (96.3%) and stem cell antigen-1 (Sca-1) (78.8%) most abundantly, followed by the hematopoietic markers CD45 (68.8%) and CD34 (25.7%). The cell surface markers CD44 (15.8%), CD146 (22.7%), CD90 (3.9%), and CD73 (0.6%) were expressed on a subset of these cells, while very low expression of the EC markers CD31 (2.0%), CD105 (0.2%), and vascular endothelial growth factor receptor 2 (VEGFR2) (0.09%) was observed (Fig 2A and 2B). Importantly, the cell surface marker profile of uterine GFP^+^ cells was markedly different from circulating GFP^+^ cells in the peripheral blood (which were mostly CD45^+^CD29^−^Sca1^−^) (S3B Fig) but was very similar to resident uterine decidual GFP^−^ cells, which were mostly CD29^+^Sca1^+^CD45^−^ (Fig 2B), suggesting that the decidual BM-derived CD45^−^ progenitors became phenotypically and functionally similar to resident decidual cells.

**Fig 3:**
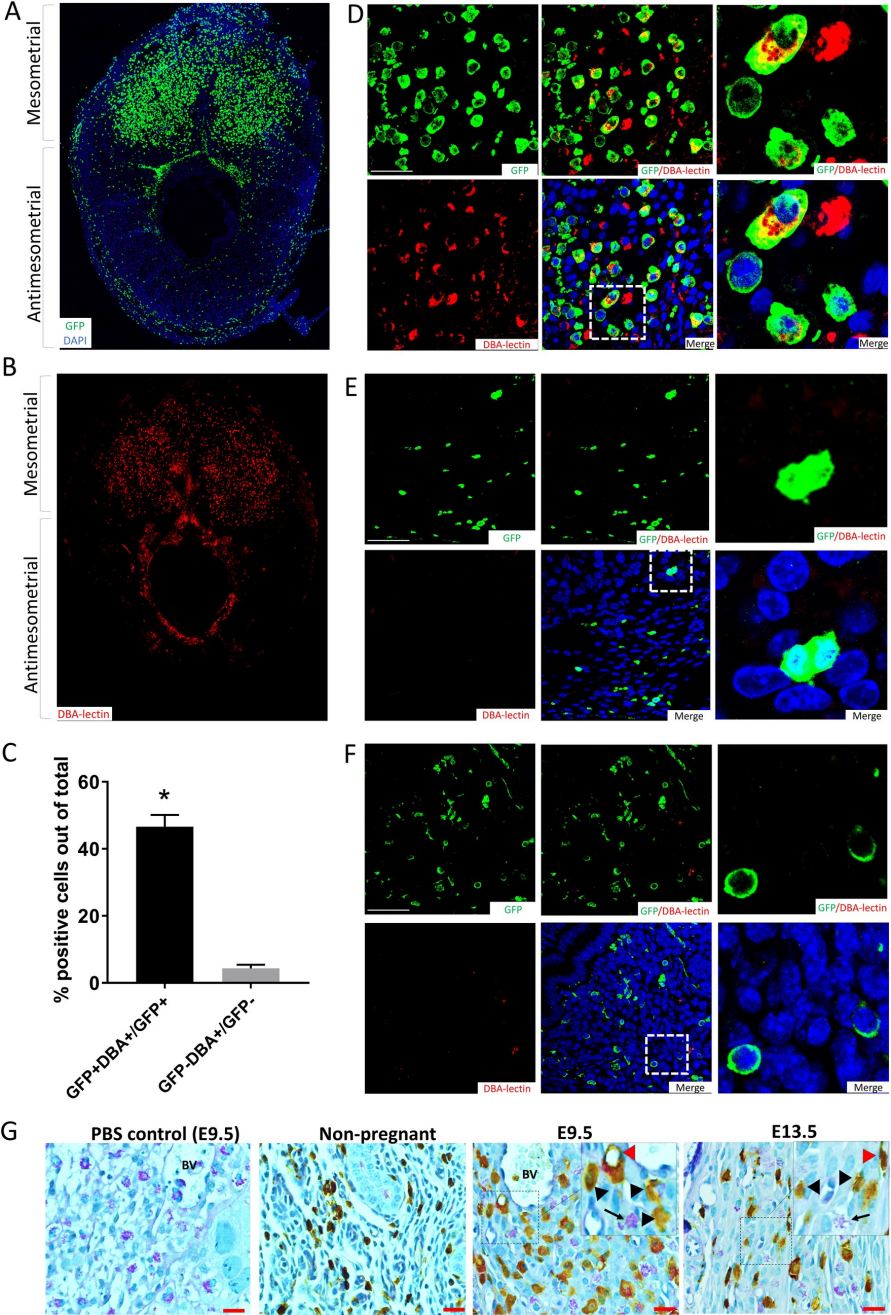
Co-staining of BMDCs with NK cell markers. (A and B) Low-magnification immunofluorescence photomicrographs of E9.5 decidua, showing (A) GFP^+^ BMDCs (green) found predominantly in the mesometrial side but also on the antimesometrial side, while (B) DBA-lectin-positive NK cells (red) are found exclusively on the mesometrial side. (C) Quantification of DBA surface marker on BM-derived (GFP^+^) cells and non–BM-derived (GFP^−^) cells on E9.5 (*n* = 4). (D-F) Higher-magnification immunofluorescence photomicrographs of (D) E9.5 mesometrial decidua, (E) antimesometrial decidua, or (F) nonpregnant mice uteri sections demonstrating co-staining of a subset of GFP^+^ BMDCs (green) with NK cell–specific stain DBA-lectin (red). Sections were counterstained with DAPI showing nuclei (blue). The images on the right of each panel are higher magnification of the corresponding dashed areas. (G) Uterine sections from nonpregnant, E 9.5, or ED 13.5 pregnant mice co-stained with PAS (purple) and GFP antibody (brown) showing PAS^+^ NK cells (black arrow), BM-derived NK cells (red pointer) and other non-NK BMDCs (black pointer). Inset shows a higher magnification of the same photomicrograph. Scale bar, 20 μm. Bar graphs represent mean ± SEM. *p < 0.0001. Underlying data are available in S1 Data. BM, bone marrow; BMDC, BM-derived cell; DBA, Dolichos biflorus agglutinin; GFP, green fluorescent protein; NK, natural killer; PAS, Periodic Acid Schiff.

Because NK cells followed by macrophages are the most abundant immune cells in the decidua during pregnancy, and some of these immune populations may not express CD45, we further confirmed the nonhematopoietic nature of the majority of GFP^+^CD45^−^ cell population using flow cytometry for other immune/hematopoietic markers as well as specific immunostaining. As some populations of uterine NK cells do not express NK1.1 [26], we used CD335 as an additional NK cell marker because it is known to be uniformly expressed on all NK cell subsets [27]. Flow cytometry analysis showed that the vast majority of GFP^+^CD45^−^ cells (>95%) were negative for the NK markers NK1.1, CD335, macrophage marker F4/80, as well as other hematopoietic markers (CD34 and CD44) (Fig 2C and 2D), indicating their nonhematopoietic nature. Moreover, the beta1 integrin cell surface receptor CD29, which is highly expressed on decidual cells [28], was found on the majority of this putative stromal decidual cell population (GFP^+^CD45^−^) and expressed on a significantly higher proportion of cells than uterine GFP^+^CD45^+^ cells (BM-derived leukocytes) (Fig 2C and 2D). As expected, uterine BM-derived leukocytes (GFP^+^CD45^+^) demonstrated high expression of typical immune (NK1.1, CD335, F4/80) and hematopoietic markers (CD34, CD44) (Fig 2C and 2D). Immunofluorescence analysis of implantation site sections on E9.5 confirmed that many GFP^+^ cells were Sca1^+^ but CD45^−^ and CD31^−^ and located in the decidual stroma, indicating that they were stromal cells and not hematopoietic or ECs (Fig 2E–2G). Dolichos biflorus agglutinin (DBA) lectin is the commonly used marker for mouse uterine NK cells as it stains both cytoplasmic granules as well as the cell membrane, thus identifying also immature, agranular NK cells. As expected, DBA immunofluorescence co-staining demonstrated specific localization of NK cells only on the mesometrial side of the implantation site (Fig 3B). GFP^+^ BMDCs were abundant on the mesometrial side (Fig 3A), where 46.6% of GFP^+^ cells were found to be DBA^+^ NK cells (Fig 3C–3F). However, GFP^+^ BMDCs were also found on the antimesometrial side, indicating that the majority of GFP^+^ cells were not NK cells. Periodic Acid Schiff (PAS) reagent is another NK-specific marker staining cytoplasmic granules of uterine NK cells. As some populations of NK cells are DBA negative but PAS^+^, PAS has been shown to be more inclusive of all decidual NK cell populations [29]. Co-immunostaining with PAS corroborated our findings that the majority of GFP^+^ cells were not NK cells (Fig 3G). In addition, immunostaining with F4/80 macrophage marker identified some GFP^+^ to be macrophages (S5 Fig), consistent with our flow cytometry data.

To confirm the decidual stromal identity of GFP^+^ cell population subsets, we assessed expression of decidual-specific markers. Decidual prolactin-related protein (DPRP) is a pregnancy-specific protein produced and secreted exclusively by decidual stromal cells (DSCs) [30], which is first detected on the antimesometrial side after day 5, followed by the mesometrial side from day 8 onwards [30]. While DPRP expression was absent in the nonpregnant uterus and on E5.5 (prior to decidualization), up to 29.5% of GFP^+^ cells in the decidua were positive for DPRP on E9.5 and thereafter, indicating that a substantial population of BMDCs recruited to the uterus had differentiated into functional prolactin-producing decidual cells (Fig 2H and 2I). Remarkably, the proportion of DPRP^+^ cells in the decidua was greater in the GFP^+^ population versuss the non-GFP resident cells throughout pregnancy on E9.5 (29.5% versus 12.8%), and nonsignificantly increased on E13.5 (22% versus 14.0%) and E17.5 (22.2% versus 17.0%) (Fig 2I), indicating that, compared with the resident cells, recruited BMDCs in decidua differentiated preferentially into decidual prolactin-immunoreactive cells. Moreover, we performed immunostaining for progesterone receptor (PR), a nuclear receptor that is specifically expressed on DSCs without expression in uterine immune cells [31] and is considered a hallmark of decidualized stromal cells. Interestingly, the percentage of GFP^+^ cells acquiring PR expression was lower than in the GFP^−^ cell population but followed the same trend of progressive increase to mid-gestation, reaching up to approximately 30% PR positivity on E9.5 and E13.5 (Fig 2J–2M), further corroborating their decidual stromal identity. To further establish the decidual stromal phenotype of GFP^+^ BMDC cell subsets, we performed single-cell RNA sequencing (scRNAseq) analysis of the E9.5 mouse implantation site, timing of peak BMDCs, using the same GFP BMT model. Differential gene expression analysis of a total of 7,275 cells single cells guided by established markers identified the major clusters of immune cells, DSCs, fibroblasts (FBs), and ECs (S6A Fig). A sub-analysis of immune cells and DSCs by graph-based clustering method identified five clusters of DSCs visualized by Uniform Manifold Approximation and Projection (UMAP) (S6B Fig). Importantly, GFP^+^ BMDCs were found within all five DSC clusters (S6C Fig), and GFP^+^ cells comprised from 1.6% to 15.9% of total DSCs. The GFP^+^ BMDCs within the DSC clusters were found to differentially express multiple DSC markers, including DPRP (Prl8a2), Hoxa10, IGFBP-4, BMP-2, Hand2, collagen isoforms, and others (S6D Fig), in contrast to the immune cells. Notably, there was variability in expression of some decidual stromal markers between the five different DSC clusters. This is consistent with a recent scRNAseq study of human decidua that demonstrated graded expression of many of the DSC markers in DSCs along the DSC trajectory [32]. Within each DSC cluster, GFP^+^ BMDCs displayed a similar expression profile of DSC markers to the GFP^−^ resident DSCs (S6D Fig). Overall, expression of proliferation markers Mki67 and Pcna was similar between immune and DSCs clusters (S7A and S7B Fig). Among GFP^+^ BMDCs, no significant differences were found in expression of the proliferation markers Mki67 and Pcna between immune and DSC subsets (S7C Fig). The recruitment of nonhematopoietic BMDCs to decidua during implantation and early pregnancy and their stromal decidual phenotype suggested that these cells may play an important role during pregnancy.

### BMDCs do not fuse with host decidual cells

Cell fusion is known to occur between BMDCs and cancer cells at variable frequencies [33]. Cell fusion between BMDCs and DSCs can potentially lead to misidentification of GFP^+^ BMDCs as stromal cells. To investigate whether cell fusion occurs between BMDCs and host decidual cells, we utilized the well-established Cre-Lox P recombination approach [34, 35] with a dual color fluorescent mT/mG system. In this system, prior to Cre recombination, cell membrane tdTomato (mT) is expressed. Upon Cre recombinase expression in the cell, membrane enhanced green fluorescent protein (EGFP) (mG) fluorescence expression replaces the mT expression. mT/mG mice co-expressing Cre recombinase transgene ubiquitously under β-actin-Cre promoter were used as positive controls, confirming the efficiency of Cre-mediated conversion from mT to mG in the blood as well as uterus in this system (S8 Fig). mT/mG transgenic mice were used as BM donors. BMT was performed following the same 5-FU–based BMT regimen into β-actin-Cre mice. The BM transplants from mT/mG donor into WT mice served as negative controls. Flow cytometry demonstrated that only BMDCs expressing mT and not mG were found at the implantation site on E9.5 (S8 Fig), timing of peak BMDCs recruitment, in both Cre as well as WT control mice. This indicates that the nonhematopoietic phenotype of decidual BMDCs is not explained by cell fusion with resident decidual cells.

### MSCs are mobilized to circulation during pregnancy and undergo decidualization in vitro

Following our observations that adult BMDCs give rise to stromal decidual cells in our BMT model, we wished to investigate whether GFP^+^ BM MSCs, the putative source of BM-derived DSCs, are reconstituted in BM of the transplanted mice following our non-gonadotoxic 5-FU–based submyeloablation protocol. Irradiation has been shown to be associated with severe and permanent damage of BM stromal cells, limiting engraftment of transplanted hematopoietic stem cells (HSCs) and MSCs [36], while 5-FU preconditioning results in a transient damage to the BM stromal cell niche, followed by extensive remodeling of MSCs in the BM supporting donor cell engraftment [37]. For this, we extracted BM from mice that underwent BMT from GFP donor according to our model. Samples were gated on Lineage (Lin)^−^/Sca-1^+^/CD45^−^ to define MSCs using a broad definition, consistent with prior works [38,39]. Flow cytometry analysis of BM cells showed the presence of putative MSCs within the transplanted GFP^+^ cell population (GFP^+^Sca1^+^Lin^−^CD45^−^) (S2A Fig). GFP^+^ BM MSCs accounted for 0.01% of total BM cells and were less abundant than GFP^+^ HSCs (GFP^+^Sca1^+^Lin^−^CD45^+^, 0.19%). GFP^+^ MSCs were also found in peripheral blood, albeit at much lower frequency (0.002%) (S3A Fig). We cultured the BM cells and further analyzed these GFP^+^ cells for the three established criteria for definition of multipotent mesenchymal stromal cell. BM GFP^+^ cells were adherent to plastic and were able to grow in culture conditions supporting MSC expansion (S2C Fig); these cultured GFP^+^ cells were analyzed for the presence of multiple MSC markers by flow cytometry, demonstrating that a fraction of GFP^+^ cells co-express established MSC markers Sca-1, CD29, and CD44 (S2B Fig). Cultured BM GFP^+^ cells were shown to have trilineage differentiation ability in vitro to adipogenic, osteogenic, and chondrogenic lineages (S2D–S2F Fig). Taken together, these data indicate engraftment of GFP^+^ MSC populations in the BM of the 5-FU BMT model. In addition, these cultured BM cells were treated with established decidualization agents medroxyprogesterone acetate (MPA) and/or 8-bromo-cAMP (cAMP), and their ability to undergo decidualization in vitro was evaluated. Cultured BM cells showed characteristic decidual morphological changes from elongated shape (control) to broad hexagonal shape in response to decidualization stimuli, which was most prominent in the combined cAMP+MPA treatment group (S2G Fig). Moreover, expression of DPRP mRNA (Prl8a2), a specific marker of decidualization in the mouse [40, 41], was up-regulated on days 8 and 14 of culture in the BM cells subjected to decidualization treatment (S2H Fig). Taken together, decidualization of BM MSCs demonstrated in vitro is consistent with our in vivo observations of BMDC transformation to DSCs.

Next, we wished to explore the dynamics of BM-derived MSCs and HSCs in BM and circulation during mouse pregnancy. Flow cytometry analysis was performed on BM and peripheral blood from nonpregnant as well as pregnant E5.5 and E9.5 mice, timing of peak BMDCs recruitment to the uterus. Circulating MSCs were found to be significantly increased in the circulation of pregnant mice from 0.002% in the nonpregnant state to 0.007% on E5.5, and further increased to 0.014% on E9.5 (approximately 7-fold compared with nonpregnant) (S9A Fig). In contrast, circulating HSCs remain unchanged from nonpregnant through E9.5 (S9A Fig). BM MSCs and HSCs were not significantly different between nonpregnant and pregnant E9.5 (S9B Fig). This evidence indicates that BM MSCs are increasingly mobilized to the circulation during pregnancy, supporting our findings of increased numbers of BM-derived CD45^−^ cells in the pregnant uterus.

### BMDCs from WT donors rescue pregnancy loss in subfertile heterozygous Hoxa11^+/−^ mice

To investigate the functional importance of the nonhematopoietic contribution of BMDCs to implantation and maintenance of pregnancy, we took advantage of the Hoxa11^−/−^ (KO) and Hoxa11^+/−^ mouse models. Homeobox-containing (Hox) genes are developmentally regulated transcription factors belonging to a multigene family. Among these, Hoxa10 and Hoxa11 are crucial for reproductive tract development, endometrial growth and differentiation, and embryo implantation by mediating some functions of sex steroids [42–46]. Hoxa11 KO female mice have abnormally small uteri characterized by stromal atrophy and absence of glands and are sterile due to an endometrial receptivity defect [42,47]. Upon mating with WT males, they form embryos but fail to mount a decidual reaction, leading to implantation failure [42,47]. In Hoxa11^+/−^ mice, implantations occur but are characterized by increased resorptions (pregnancy loss) and, ultimately, reduced litter sizes [42]. In our experience, Hoxa11^+/−^ mice also had reduced litter sizes (mean 5.4, *n* = 78) compared with WT mice (mean 8.3, *n* = 96). Based on our observation of a significant contribution of BMDCs to nonhematopoietic cellular content of the decidual stroma, we hypothesized that BMT from a WT mouse may rescue the reproductive defects in these mice. We chose the Hoxa11-deficient mouse model for our investigations because it was shown that Hoxa11 expression in the BM is restricted to mesenchymal stem/stromal cell progenitors and it is not expressed in hematopoietic cells [25], thus allowing us to interrogate the role of nonhematopoietic BMDC contribution to pregnancy. Using Hoxa11^+/−^ mice, in which the Hoxa11 gene is replaced by EGFP knock-in (Hoxa11-GFP), we performed flow cytometry, which confirmed the absence of Hoxa11 expression in hematopoietic cells of BM, spleen, and peripheral blood and expression of Hoxa11 in a small subset of nonhematopoietic (CD45^−^) tibial BM cells (S10 Fig), consistent with its regional-restricted expression in the zeugopod [25]. Hoxa11 was abundantly expressed in the uterine implantation site of these mice exclusively in nonhematopoietic (CD45^−^) cells. To confirm the ability of nonhematopoietic BMDCs to migrate to the uterus and contribute to nonhematopoietic decidual Hoxa11^+^ cell population, we analyzed WT mice transplanted with BM from Hoxa11-GFP donors using the same 5-FU–based submyeloablation regimen. Multicolor flow cytometry analysis of the E9.5 implantation site of transplanted mice showed the presence of BM-derived GFP^+^Hoxa11^+^ cells, which were all CD45 negative in addition to being negative for NK and myeloid markers NK1.1 and CD11b, respectively (S11A and S11B Fig). These GFP^+^Hoxa11^+^ cells accounted for 0.5% of total cells in the implantation site. In contrast, Hoxa11^+^GFP^+^ cells were not found in nonpregnant uterus (S11A and S11B Fig).

**Fig 4:**
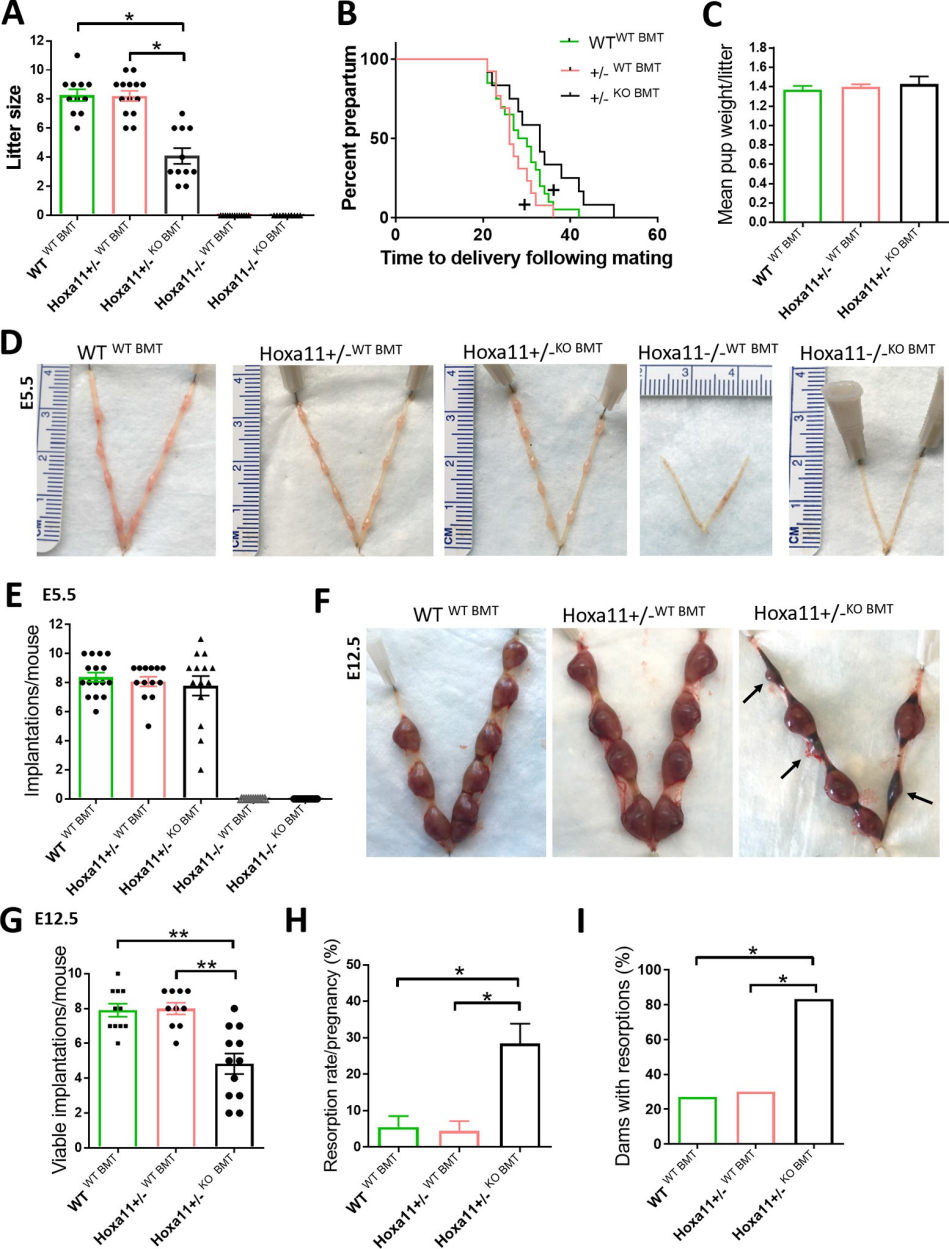
BM transplantation from WT donors prevents pregnancy loss in Hoxa11^+/−^ mice. BMTs were performed from WT into Hoxa11^+/−^ (Hoxa11^+/−WT BMT^), WT into HoxA11^−/−^ (Hoxa11^−/−WT BMT^), Hoxa11^−/−^ into Hoxa11^+/−^ (Hoxa11^+/−KO BMT^), and HoxA11^−/−^ into Hoxa11^−/−^ (Hoxa11^−/−KO BMT^). WT mice receiving BMT from WT donors (WT^WT BMT^) served as controls. (A-C) Effect of BMT from different genotypes on (A) litter size, (B) time to delivery, and (C) mean pup weight per litter following breeding (*n* = 11–15/group). *p < 0.01. +*p* < 0.05 versus Hoxa11^+/−KO BMT^. (D and E) Representative uterine images (D) and quantitation of mean number of implantations per mouse on E5.5 (E) in WT, Hoxa11^+/−^, and HoxA11^−/−^ mice that received BMT from either WT or KO donors (*n* = 10–16/group). (F-I) Pregnancy resorptions on ED 12.5 in WT and Hoxa11^+/−^ mice that received BMT from either WT or KO donors (*n* = 10–12/group). (F) Representative images of uteri showing resorption sites (black arrows). (G) Mean number of viable implantations per mouse. (H) Resorption rate per mouse pregnancy. (I) Percentage of dams with at least one resorption. In all panels, bar graphs represent mean ± SEM. *p ≤ 0.05, **p ≤ 0.01. Underlying data are available in S1 Data. BM, bone marrow; BMT, BM transplant; Hoxa11, Homeobox a11; KO, knockout; WT, wild-type.

KO and Hoxa11^+/−^ female mice underwent BMT from either WT or KO mice following the same 5-FU–based submyeloablation protocol. WT mice undergoing BMT from WT donors served as controls. First, we examined the reproductive performance of the mice following a 1-month harem breeding trial. Heterozygous Hoxa11^+/−^ mice receiving BMT from WT mice (Hoxa11^+/−WT BMT^) had comparable mean litter size to WT^WT BMT^ animals and had significantly greater mean litter size, as compared with Hoxa11^+/−^ mice receiving KO BMT (Hoxa11^+/−KO BMT^) (8.2 versus 8.3 versus 4.0, respectively) (Fig 4A). The KO mice receiving either BMT treatment delivered no litters nor had any visible signs of pregnancy (Fig 4A). While overall pregnancy rates were the same in the WT and Hoxa11^+/−^ groups, differences were noted in time to conception. The Hoxa11^+/−WT BMT^ mice had comparable time to conception with WT^WT BMT^ control animals but shorter time to conception as compared with Hoxa11^+/−KO BMT^ mice (Fig 4B). No differences were noted in mean pup weight per litter between the various groups (Fig 4C).

Next, we investigated whether the observed difference in litter size following treatment was related to early pregnancy (implantation) and/or later pregnancy (resorptions) events. To this end, timed pregnancies following the same treatment protocols were ended either on E5.5 or E12.5 for assessment of implantation and resorption, respectively. On E5.5, there were no differences in the number of implantations between the Hoxa11^+/−^ mice receiving either WT or KO BMT, and the control WT mice receiving WT BM genotype. In contrast, no distinct implantation sites were noted in the KO mice receiving either treatment (Fig 4D and 4E). On E12.5, heterozygous Hoxa11^+/−WT BMT^ mice showed a decreased resorption rate (4.4%) versus Hoxa11^+/−KO BMT^ mice (28.4%) and had a comparable resorption rate to that of WT controls (5.5%) (Fig 4F and 4H). Similarly, the mean number of viable implantations in Hoxa11^+/−WT BMT^ mice was greater than Hoxa11^+/−KO BMT^ but the same as WT control mice (8.0 versus 4.9 versus 7.9, respectively) (Fig 4G). Furthermore, the percentage of dams with resorptions was greater in the Hoxa11^+/−KO BMT^ mice as compared with both the Hoxa11^+/−WT BMT^ group as well as the WT control group (83% versus 30% versus 27%, respectively) (Fig 4I). These data indicate that BMT treatment of Hoxa11^+/−^ mice from WT donors results in normalization of litter size due to prevention of pregnancy loss following implantation.

### Hoxa11-expressing BMDCs induce expression of known decidualization genes in the implantation site to rescue pregnancy

**Fig 5:**
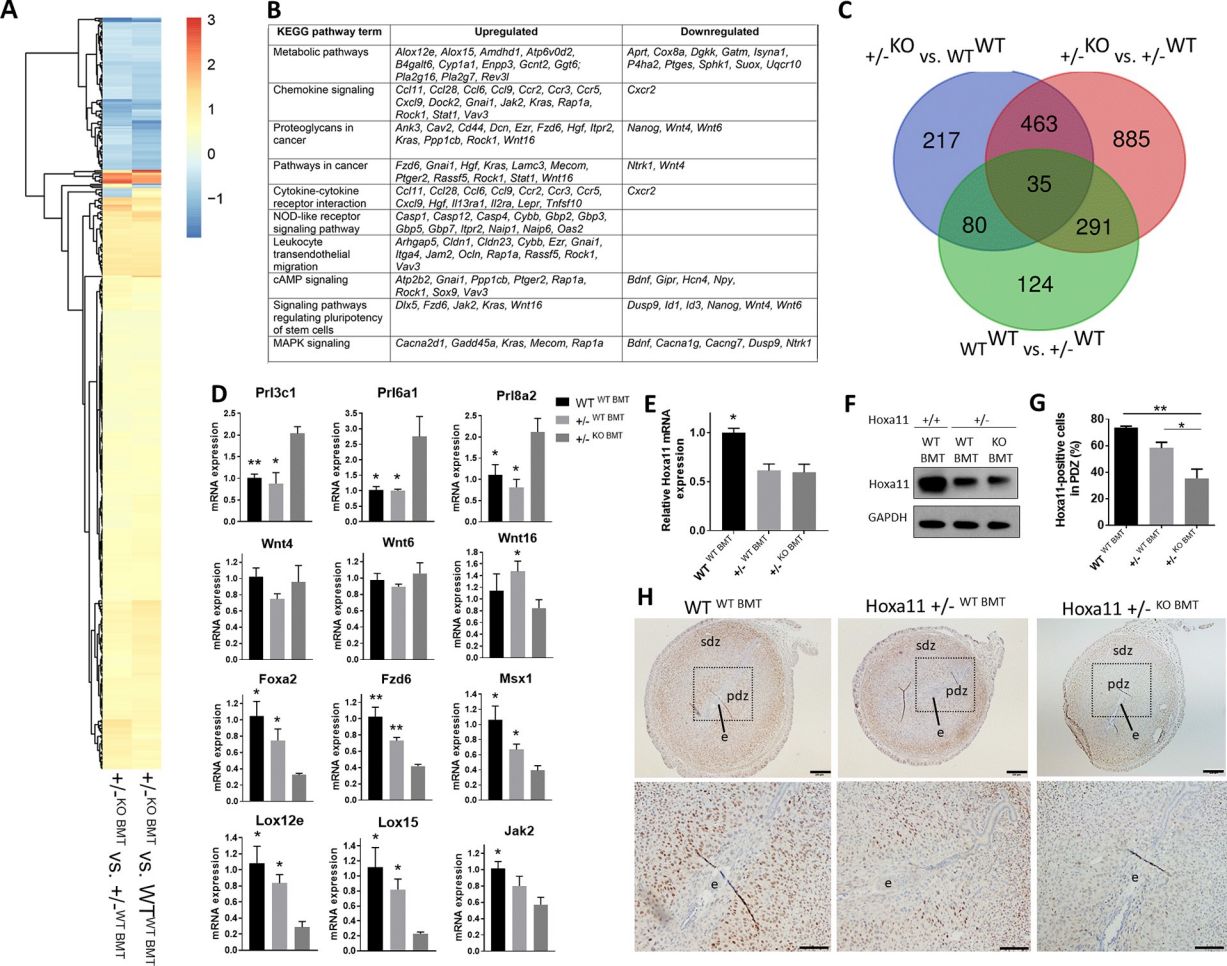
BM transplantation from WT donors normalizes the uterine transcriptome of Hoxa11^+/−^ mice. (A-C) RNA-seq analysis of implantation sites on E5.5 in Hoxa11^+/−WT BMT^, Hoxa11^+/−KO BMT^, and WT^WT BMT^. (A) Heat map of the expression fold change of 498 commonly DEGs in the comparisons of Hoxa11^+/−KO BMT^ versus Hoxa11^+/−WT BMT^, and Hoxa11^+/−KO BMT^ versus WT^WT BMT^. (B) The top 10 KEGG pathways enriched in genes commonly differentially expressed in the comparisons of Hoxa11^+/−KO BMT^ versus Hoxa11^+/−WT BMT^, and Hoxa11^+/−KO BMT^ versus WT^WT BMT^. (C) Three-way Venn diagram of the comparisons of Hoxa11^+/−KO BMT^ versus Hoxa11^+/−WT BMT^, Hoxa11^+/−KO BMT^ versus WT^WT BMT^, and Hoxa11^+/−WT BMT^ versus WT^WT BMT^. (D) RT-PCR validation of gene expression of select genes from the 498 commonly DEGs that have important roles in decidualization. *p ≤ 0.05 and **p ≤ 0.01 versus Hoxa11^+/−KO BMT^ (*n* = 3–5/group). (E-H) Hoxa11 expression in implantation sites on ED5.5 in Hoxa11^+/−WT BMT^, Hoxa11^+/−KO BMT^, and WT^WT BMT^ (*n* = 5–6/group). (E) Hoxa11 mRNA expression and (F) Hoxa11 protein expression in ED5.5 implantation sites. (G and H) Sections of implantation sites from ED5.5 stained with Hoxa11 antibody (brown) (H). Inset (below) shows high magnification photomicrograph of the dashed area and the embryo (e). The primary decidual zone (PDZ) and secondary decidual zone (SDZ) are noted. (G) Corresponding quantification of Hoxa11-positive cells in the PDZ. Bar graphs represent mean ± SEM. *p ≤ 0.05, **p ≤ 0.01. Scale bars, 200 μm (upper panel), 100 μm (lower panel). See also S12 and S16 Fig. Underlying data are available in S1 Data. BM, bone marrow; BMT, BM transplant; DEG, differentially expressed gene; Hoxa11, Homeobox a11; KEGG, Kyoto Encyclopedia of Genes and Genomes; KO, knockout; PDZ, primary decidual zone; RNA-seq, RNA sequencing; SDZ, secondary decidual zone; WT, wild-type.

The maternal component of the implantation site consists of stromal cells of the uterine endometrium that undergo a tightly controlled process of decidualization upon contact with the early embryo. These stromal cells undergo proliferation and differentiation into large epitheloid decidual cells in a process that is critical to the establishment of fetal–maternal communication and pregnancy maintenance. To gain insight into the mechanism by which BMDCs lead to pregnancy loss prevention in Hoxa11^+/−^ mice, we focused on profiling gene expression by RNA-seq in the uterine tissue of the implantation site on E5.5, a time of decidualization which is critical for successful embryo implantation. To this end, we assessed expression patterns in Hoxa11^+/−WT BMT^ and WT^WT BMT^ when they were compared with Hoxa11^+/−KO BMT^. There were fewer differentially expressed genes (DEGs) in the comparison of WT^WT BMT^ versus Hoxa11^+/−WT BMT^ (530 genes), as compared with between WT^WT BMT^ versus Hoxa11^+/−KO BMT^ (795 genes) (Fig 5C). We found a total of 498 genes that were significantly commonly differentially expressed (false discovery rate [FDR]-adjusted *p*-value < 0.05) in the comparisons of Hoxa11^+/−WT BMT^ versus Hoxa11^+/−KO BMT^ and WT^WT BMT^ versus Hoxa11^+/−KO BMT^ (Fig 5A and S2 Data). Remarkably, 98.6% of the 498 commonly DEGs were either up- or down-regulated in the same direction in Hoxa11^+/−WT BMT^ and WT^WT BMT^ mice (Fig 5A and S2 Data), suggesting that BMT from WT donors normalized the uterine implantation transcriptome of Hoxa11^+/−WT BMT^ in favor of normal implantation. Interestingly, the 498 commonly DEGs were significantly enriched (*N* = 27; Fisher's exact test *p*-value = 1.29 × 10^−9^) for decidualization genes, based on a combined list of 323 genes with known role in mouse decidualization according to Mouse Genome Informatics (MGI) database and the literature [48]. Those 27 genes included key regulators of decidualization, such as prolactin superfamily members (*Prl3c1*, *Prl6a1*, *Prl8a2*) [40, 49, 50], Wingless/Integrated (Wnt) family members (*Wnt4*, *Wnt6*, *Wnt16*, *Fzd6)* [51–53], *Msx1* [54], *Ptges* [55], *Igfbp2*, *A2m* [56], and *Foxa2* [57]. We independently confirmed differential expression of selected candidates by quantitative RT-PCR using additional biological replicates (Fig 5D). Using the list of commonly differentially regulated genes for gene ontology (GO) and pathway analysis revealed that the top 10 enriched Kyoto Encyclopedia of Genes and Genomes (KEGG) pathways included many pathways known to be important for implantation such as metabolism, chemokine signaling pathway, proteoglycans in cancer, cytokine-cytokine receptor interaction, NOD-like receptor signaling pathway, leukocyte transendothelial migration, cAMP signaling pathway, signaling pathways regulating pluripotency of stem cells, and MAPK signaling pathway (Fig 5B). In addition, major canonical pathways enriched in the Ingenuity Pathway Analysis (IPA) included pathways known to play important roles in implantation such as leukocyte extravasation, planar cell polarity (PCP) pathway, eicosanoid signaling, chemokine signaling, mouse embryonic stem cell pluripotency, regulation of epithelial-mesenchymal transition, and WNT/β-catenin (S12 Fig).

Uterine Hoxa11 mRNA and protein expression on implantation day (E5.5) was greater in WT^WT BMT^ mice as compared with Hoxa11^+/−KO BMT^ and Hoxa11^+/−WT BMT^ mice, but similar between the two latter groups (Fig 5E and 5F). However, close examination of the Hoxa11 immunostaining pattern in uterine sections revealed that Hoxa11-positive cells were more abundant in the primary decidual zone (PDZ) surrounding the embryo in Hoxa11^+/−WT BMT^ as compared with Hoxa11^+/−KO BMT^ (Fig 5G and 5H). In addition, WT^WT BMT^ control mice showed greater abundance of Hoxa11-expressing cells around the implantation site as compared with both Hoxa11^+/−^ mice groups (Fig 5G and 5H). No histological differences were noted in implantation sites between Hoxa11^+/−^ mice WT BMT versus KO BMT. Taken together, these data suggest that BMDCs can affect global gene expression at the implantation site, favoring pregnancy maintenance and ultimately leading to rescue of pregnancy losses arising from endometrial stromal defects.

It was shown that triple mutant mice with simultaneous KO of Hoxd9, Hoxd10, and Hoxd11 have increased number of leukocytes and altered proportions of immune cell in the uterus, with increase in myeloid lineage populations of macrophages and granulocytes [58]. To evaluate whether Hoxa11 deficiency affects uterine leukocyte populations or recruitment of BMDCs to the uterus, flow cytometric analysis was performed on nonpregnant or pregnant uteri of KO and Hoxa11^+/−^ mice, respectively, which underwent BMT from transgenic tdTomato-expressing BM donors. WT mice that underwent BMT from tdTomato donors served as controls. No differences were found in numbers of leukocytes or in numbers of BMDCs recruited to the nonpregnant uteri of KO mice or the implantation site of pregnant Hoxa11^+/−^ mice, as compared with WT controls (S13 Fig). In addition, no difference in the total number of BM or splenic cells was found between nonpregnant KO and WT mice, and between pregnant Hoxa11^+/−^ and WT mice.

To investigate the possibility that immune cell populations are altered in Hoxa11^+/−^ pregnancy as an underlying reason for their reproductive phenotype, we compared the immune populations in the implantation site between pregnant Hoxa11^+/−^ and WT mice (S11 Fig). No differences were found in the total number of leukocytes or in proportions of NK1.1^+^ (NK) cells, F4/80 macrophages, total CD3^+^ T cells, CD4^+^CD25^+^ T regulatory (Treg) cells, or Ly6G^+^ granulocytes in the implantation site between the two groups (S14 Fig). Taken together, these data suggest that BMDCs recruitment to the uterus and uterine immune cell populations are unaltered in Hoxa11^+/−^ mice, and it is unlikely that the rescue effect of WT BMT on resorptions in Hoxa11^+/−^ mice is mediated by the transplanted immune cell populations.

### Hoxa11-expressing BMDCs induce endometrial regeneration, including gland formation, in Hoxa11^−/−^ null mice

**Fig 6:**
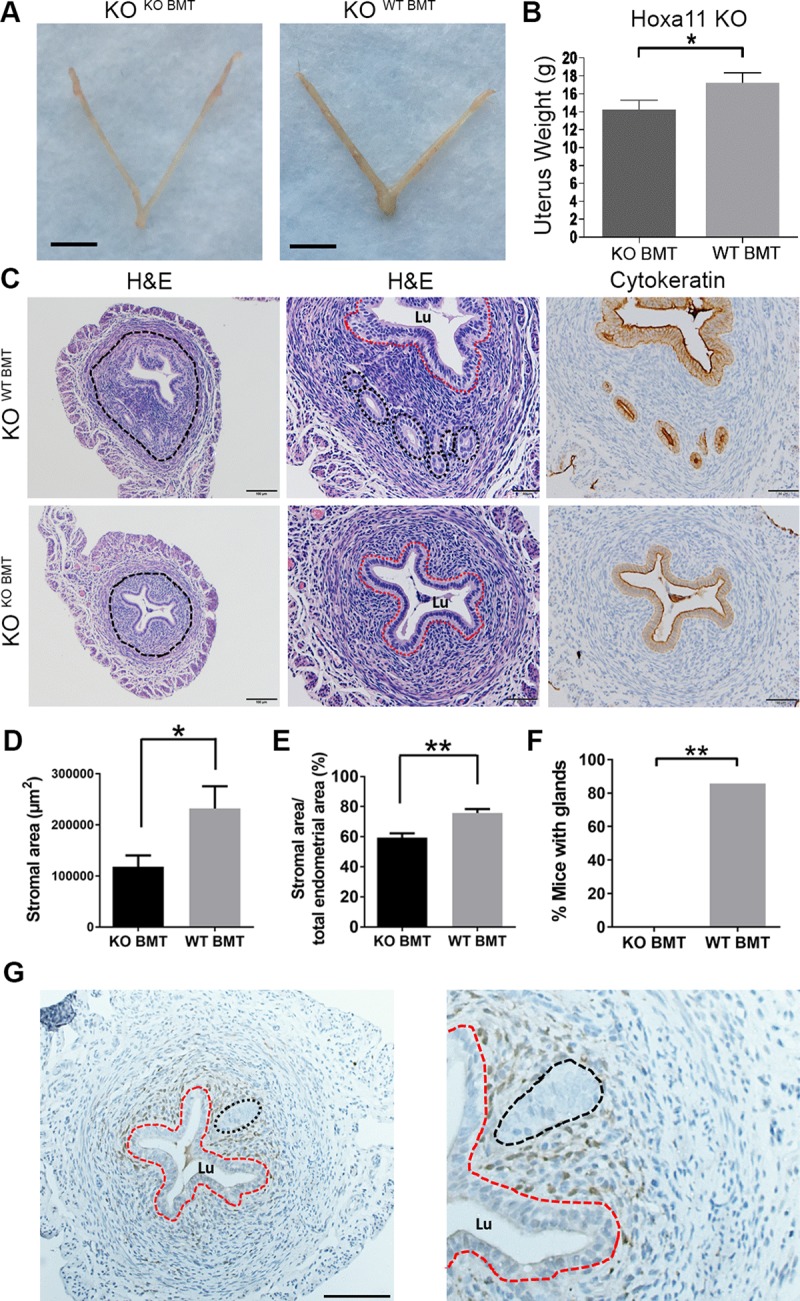
BM transplantation from WT donors leads to stromal expansion and induces glandular formation in HoxA11-null mice. (A and B) Uterus photographs (A) and mean uterine weight (B) of nonpregnant KO^WT BMT^ and KO^KO BMT^ mice. (C) HE and cytokeratin immunostaining (brown) of uterine sections from KO^WT BMT^ and KO^KO BMT^ mice. The endometrial area is encircled in the left panel and shown at higher magnification in the middle (HE) and right (cytokeratin) panels. Glands are seen only in KO^WT BMT^ mice and are surrounded by black dashed circles in the middle panel, corresponding to the cytokeratin-positive (brown) areas in the right panel. The luminal epithelium (Lu) is encircled by a red dash. Scale bars, 100 μm (left) and 50 μm (middle and right panels). (D-F) Quantification of total stromal area (μm^2^) (D), percent stromal area out of endometrial area (E), and percent mice with endometrial glands (F) in KO^WT BMT^ and KO^KO BMT^ groups. *n* = 6–7/group. (G) GFP immunostaining of uterine sections from KO^WT BMT^ showing localization of GFP^+^ BMDCs (brown) in the stroma. The Lu) is encircled by a red dash and the gland is encircled by black dash. Bar graphs represent mean ± SEM. *p ≤ 0.05, **p ≤ 0.01. See also S15 Fig. Underlying data are available in S1 Data. BM, bone marrow; BMDC, BM-derived cell; BMT, BM transplant; GFP, green fluorescent protein; HE, hematoxylin–eosin; Hoxa11, Homeobox a11; KO, knockout; Lu, luminal epithelium; WT, wild-type.

Hoxa11^−/−^ mice have abnormally small uteri characterized histologically by minimal endometrial stroma and complete absence of glands [42,47]. To evaluate whether Hoxa11-expressing BMDCs can repair the endometrial defects of KO mice, BMT was performed into KO mice either from WT or KO donors. Examination of the nonpregnant uteri of KO animals 1 month following BMT showed a significant increase in uterine weight of KO^WT BMT^ compared with KO^KO BMT^ mice (Fig 6A and 6B). This was associated histologically with profound endometrial stromal expansion in the KO^WT BMT^ group as compared with KO^KO BMT^, noted in all estrus phases (Figs 6C–6E and S15). Furthermore, gland formation was seen only in the KO^WT BMT^ group (Fig 6C and 6F). BM transplantation from GFP (WT) donors into KO mice revealed the presence of GFP^+^ BMDCs in the uterine stroma but not in endometrial glands or luminal epithelial cells (Fig 6G). These data indicate that endometrial glandular regeneration in Hoxa11^−/−^ mice is likely induced by BMDCs paracrine-mediated mechanisms rather than direct cellular contribution of BMDCs to glandular or luminal epithelium.

### Hoxa11-expressing BMDCs induce decidual reaction in Hoxa11^−/−^ null mice

**Fig 7:**
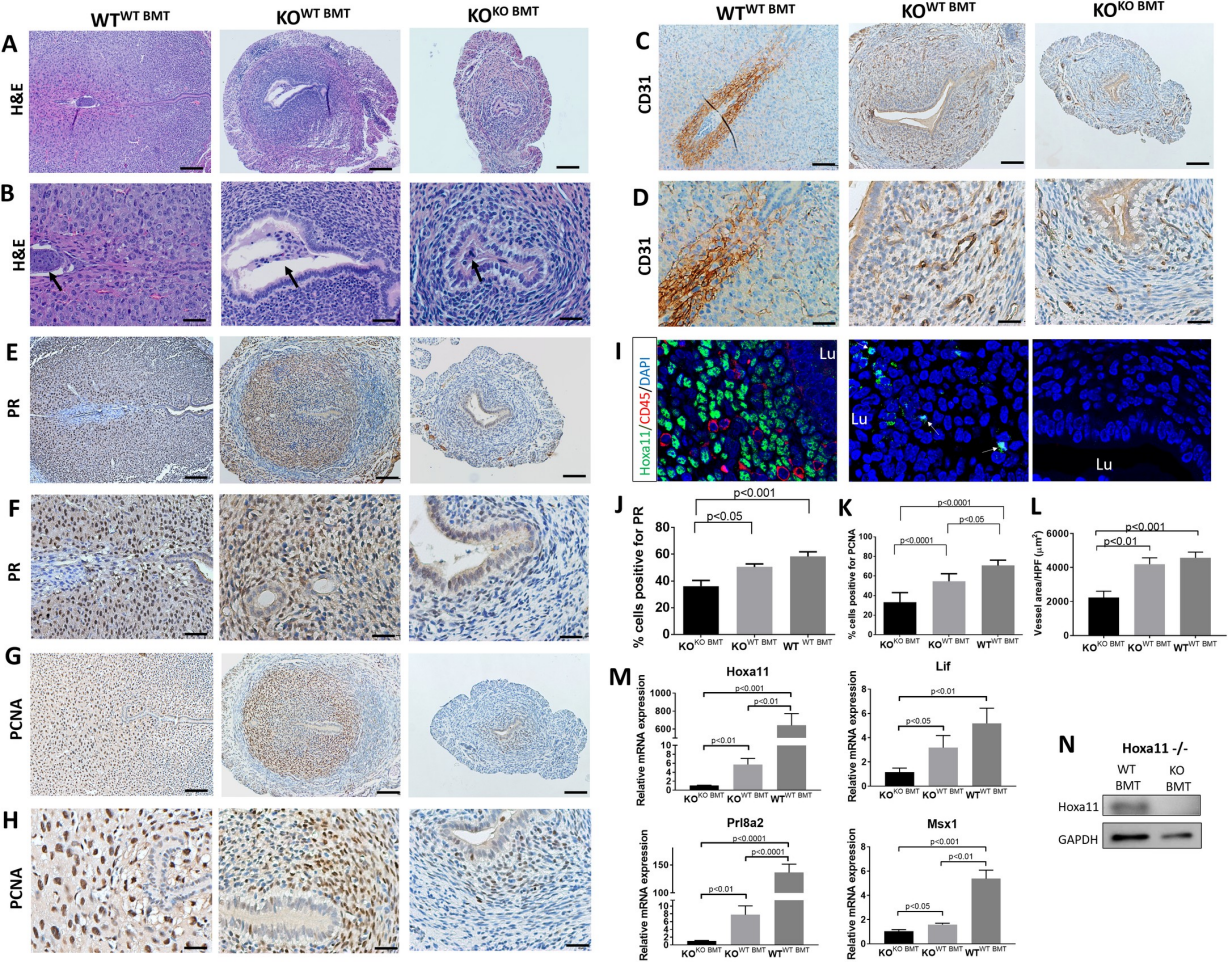
BM transplantation from WT donors leads to decidualization reaction in Hoxa11-null mice. (A-H) Sections of E5.5 implantation sites from WT^WT BMT^, KO^WT BMT^, and KO^KO BMT^ stained with HE (A and B), CD31 (C and D), PR (E and F), and PCNA (G and H). B, D, F, and H are higher magnification photomicrographs of A, C, E, and G, respectively. Black arrows point to embryos. (J, K and L) Quantification of pecent PR-positive cells (J), percent PCNA-positive cells (K), and mean blood vessel luminal area (μm^2^) in the endometrium (*n* = 3–4 mice/group). (I) Sections of E5.5 implantation sites from WT^WT BMT^, KO^WT BMT^, and KO^KO BMT^ co-stained with Hoxa11 (green), CD45 (red), and counterstained with DAPI (blue). Arrows point to Hoxa11-expressing cells. (M) Relative uterine mRNA expression of HoxA11 and implantation-related genes Prl8a2, Lif, and Msx1 normalized to GAPDH in WT^WT BMT^ versus KO^WT BMT^ versus KO^KO BMT^ on E5.5 (*n* = 4–5 mice/group). (N) Uterine Hoxa11 protein expression in KO^WT BMT^ versus KO^KO BMT^ on E5.5. Bar graphs represent mean ± SEM. *p*-values are noted on the graphs. Scale bars, 100 μm (A, C, E, G), 50 μm (B, D, F, H). Underlying data are available in S1 Data. BM, bone marrow; BMT, BM transplant; GAPDH, glyceraldehyde 3-phosphate dehydrogenase; HE, hematoxylin–eosin; Hoxa11, Homeobox a11; KO, knockout; PCNA, proliferating cell nuclear antigen; PR, progesterone receptor; WT, wild-type.

Despite lack of normal implantation sites in the Hoxa11 KO mice, areas of increased swelling and vascularization in the uterus were observed on E5.5 only in the group that received WT BMT, suggesting that some uterine reaction to the embryo has taken place (Fig 3D). Histological analysis of uterine sections on E5.5 from KO^WT BMT^ revealed significant decidual reaction. Embryos were noted attached to the uterine wall initiating the process of invasion toward the uterine stroma, which was expanded and showed characteristics of proliferation and differentiation into a decidua (Fig 7A and 7B). In contrast, KO^KO BMT^ demonstrated non-receptive uteri without any sign of decidual reaction or embryo attachment (Fig 7A and 7B). To further characterize the impact of BMT from WT donors on decidualization in KO mice, we analyzed the three hallmarks of decidualization: decidual differentiation, cell proliferation, and vascular expansion. Immunohistochemical analysis for PR, an established decidualization marker, revealed prominent expression in the decidua of KO^WT BMT^, similar in extent to the WT^WT BMT^ mice (Fig 7E, 7F and 7J). In contrast, PR expression was significantly reduced in the uteri of KO^KO BMT^, consistent with impaired decidualization. Immunostaining for PCNA (Fig 7G, 7H and 7K), a proliferation marker, demonstrated extensive cell proliferation in the KO^WT BMT^, in contrast to KO^KO BMT^ deciduae, which were largely devoid of proliferation. Finally, we looked at the impact of BMT from WT donors on decidual vascular expansion, a major prerequisite for adequate implantation. Immunostaining for ECs using CD31 (Fig 7C, 7D and 7L) revealed increased decidual blood vessel area and mean luminal area in KO^WT BMT^ versus KO^KO BMT^. Overall, uteri of Hoxa11 KO mice receiving WT BMT exhibited marked improvement in decidualization and its characteristics: stromal cell proliferation and differentiation, as well as vascular expansion. In contrast, uteri of KO mice receiving KO BMT were characterized by severe impairment of all major characteristics of decidualization, thus preventing the formation of an adequate decidual tissue.

To further investigate the underlying mechanism responsible for the decidualization effects in KO mice following WT BMT, we analyzed expression of Hoxa11 and other associated genes that are known to be essential for implantation and decidualization, such as leukemia inhibitory factor (Lif) [47], prolactin [59], and MSH homeobox 1 (Msx1) [54], in the uterus on E5.5. Hoxa11 mRNA and protein expression were observed in the uterus of KO mice receiving WT BMT, but not KO mice receiving KO BMT (Fig 7M and 7N). In addition, we found that the implantation-related genes Msx1, Prl8a2, and Lif were significantly up-regulated in the KO^WT BMT^ as compared with KO^KO BMT^ mice. Moreover, immunofluorescence analysis demonstrated the presence of Hoxa11-expressing cells in the decidua of KO^WT BMT^ but not KO^KO BMT^ mice (Fig 7I), suggesting that WT Hoxa11-expressing BMDCs were recruited to the uterus of KO mice receiving WT BMT. To exclude the possibility that the Hoxa11-expressing cells were merely circulating immune cells, we performed colocalization with the pan-leukocyte marker CD45, which demonstrated that the Hoxa11-expressing cells were nonhematopoietic (CD45-negative) stromal cells (Fig 7I). This is consistent with our flow cytometry data showing that Hoxa11 is not expressed on hematopoietic cells but is only expressed in CD45-negative nonhematopoietic cells (S10 and S11 Figs). Taken together, these data suggest that Hoxa11-expressing BMDCs can affect gene expression at the uterine implantation site, leading to profound effects on decidualization, as evidenced by promotion of decidual differentiation, cell proliferation, and angiogenesis.

## Discussion

It is well established that uterine implantation sites are areas of infiltration of many BM-derived immune cells, which play important roles at the maternal–fetal interface to promote successful pregnancy (reviewed in [6]). Adult nonhematopoietic BMDCs have been detected in both human [17–19] and mouse [18,20–23] uterine endometrium, but the use of gonadotoxic irradiation for myeloablation likely precluded pregnancy investigations using those models. Thus, the nonhematopoietic contribution of adult BMDCs to pregnant decidua as well as its potential functional importance to pregnancy has remained unknown. Here, using GFP-labeled BM cells in a non-gonadotoxic BMT mouse model, we show that implantation and early gestation are strong stimuli for adult BMDCs recruitment to the uterus, where some BMDCs acquire a decidual stromal phenotype, expressing PR, and differentiate into functional prolactin-immunoreactive decidual cells, providing a physiologic contribution to nonhematopoietic stromal decidual cell compartment. Importantly, the nonhematopoietic contribution of BMDCs to the uterus in pregnancy is strikingly different than in the nonpregnant state, as demonstrated by both our GFP BMT and Hoxa11-GFP BMT models. A recent study by Ong and colleagues using an irradiated mouse BMT model reported all BMDCs in the nonpregnant uterus to be immune cells [60]. They found that BMDCs were either positive for the pan-leukocyte marker CD45 and/or positive for the macrophage marker F4/80 [60]. Similar to these findings, in our study most BMDCs in the nonpregnant uterus were CD45^+^ immune cells (approximately 95%). However, during pregnancy the proportion of CD45^+^ cells within the BMDC population in the pregnant decidua gradually decreased, reaching a nadir (approximately 70%) at mid-gestation, whereas a substantial population of BMDCs, albeit a minority population, differentiated into nonhematopoietic decidual cells (CD29^+^CD45^−^) (approximately 30%) and expressed surface markers similar to the resident decidual cells, indicating that pregnancy drives the differentiation of BMDCs into stromal decidual cells. It was important to exclude the possibility that these CD45^−^ BMDCs were NK cells, macrophages, or other hematopoietic cells, and for this end, we utilized both flow cytometry as well as immunofluorescence with various immune and hematopoietic markers. In addition, we confirmed the identity of BM-derived stromal decidual cells by single-cell RNAseq analysis, demonstrating their clustering within DSC clusters. We also excluded the possibility that the BM-derived stromal decidual cells were a result of cell fusion.

Here, we show that a greater proportion of BMDCs are proliferating as compared with the resident decidual cells. This differential proliferation was restricted to the pregnancy period and observed at all gestational time points. The high rate of cellular division in these BMDCs suggests an important role in keeping up with the demands of rapid growth and turnover in the developing pregnant uterus. Interestingly, the proliferation rate was not found to be different between hematopoietic and nonhematopoietic BMDCs. Moreover, during pregnancy a greater proportion of BM-derived decidual cells were expressing the DPRP as compared with the resident decidual cells. DPRP is a heparin-binding cytokine that is abundantly expressed in uterine decidua and is essential for pregnancy-dependent adaptations to physiological stressors [61]. Taken together, these data suggest that the BM-derived stromal decidual cell population is functional within the developing decidua.

Over the last decade, several studies in both humans and mice have provided evidence that BMDCs give rise to various nonhematopoietic cellular compartments of the nonpregnant endometrium, including epithelial luminal, glandular, and stromal, albeit in very small numbers. These BMDCs have been suggested to play an important role in physiological endometrial tissue regeneration as well as pathologies such as endometriosis (reviewed in [62] and [63]), but their role in implantation and pregnancy maintenance has remained unknown. To study the functional nonhematopoietic contribution of BMDCs to implantation and pregnancy, we utilized two mouse models harboring endometrial stromal cell-specific defects; first, we used Hoxa11^−/−^ null mice, which have abnormally small uteri with stromal atrophy and absence of endometrial glands and are infertile due to lack of decidualization, leading to embryo implantation failure; and second, we used heterozygous Hoxa11^+/−^ mice, which have subfertility characterized by increased pregnancy resorptions and reduced litter size. We demonstrate that BMT from WT mice into syngeneic Hoxa11^−/−^ mice results in endometrial stromal expansion and gland formation, as well as marked decidualization and its characteristic hallmarks, decidual differentiation, cell proliferation, and angiogenesis, compared with the complete lack thereof in Hoxa11^−/−KO BMT^. Hoxa11-expressing stromal cells were identified at the implantation sites of Hoxa11^−/−WT BMT^ mice and were associated with increased uterine Hoxa11 mRNA and protein expression, as well as up-regulation of genes known to be crucial for implantation in the uterus, including Lif [47], prolactin [59], and Msx1 [64]. Both LIF and prolactin are known transcriptional targets of Hoxa11 [47,59], while MSX1 regulation by Hoxa11 has not been previously described. Our results suggest that Hoxa11 positively regulates, either directly or indirectly, MSX1 in the uterus. Uterine glands are critical for implantation and decidualization in the mouse due to secretion of LIF in the preimplantation period [65]. Absence of LIF or loss of endometrial glands in the mouse uterus results in implantation and decidual defects [65,66], which can be rescued in both cases by LIF administration [65,66]. Therefore, the induction of gland formation in Hoxa11^−/−^ mice following WT BMT is likely responsible for the up-regulation of LIF expression and associated decidualization effects observed in our model. In addition, WT BMT into heterozygous Hoxa11^+/−^ mice also resulted in up-regulation of LIF to levels comparable to WT (S16 Fig), suggesting that LIF up-regulation is not only due to restoration of glands but independently occurs at the time of decidualization, induced by Hoxa11-expressing BMDCs. Our findings are also consistent with the critical role of Hoxa11 in uterine stromal cell proliferation [67]. Interestingly, BMDCs were found in the uterine stroma but not in endometrial glands or luminal epithelium of Hoxa11^−/−^ mice. This suggests that endometrial glandular regeneration was induced by BM-derived stromal paracrine-mediated mechanisms rather than direct cellular contribution to glandular or luminal epithelium. Taken together, these data suggest that gene expression of uterine BMDCs leads to major downstream effects on uterine processes governing implantation.

In the present study, we show that BMT from WT donors into Hoxa11^+/−^ mice results in normalization of litter size through the rescue of pregnancy loss, as compared with Hoxa11^+/−KO BMT^. Global Hoxa11 mRNA or protein expression was not substantially different between the Hoxa11^+/−^ mice receiving WT or KO BMT, likely due to the low absolute numbers of BMDCs expressing Hoxa11, as demonstrated in our Hoxa11-GFP BMT model. However, Hoxa11 is an important transcription factor and the presence of normal Hoxa11-expressing cells, albeit at small numbers, is likely to have downstream gene expression amplification effects. Moreover, expression of Hoxa11 in the PDZ was more prominent in the Hoxa11^+/−WT BMT^ as compared with Hoxa11^+/−KO BMT^, suggesting the importance of the spatial expression pattern of this transcription factor to pregnancy maintenance. RNA-seq analysis of implantation site uterine tissues demonstrated that Hoxa11^+/−WT BMT^ and WT^WT BMT^ mice exhibited common differential expression of numerous genes when compared with Hoxa11^+/−KO BMT^, with significant enrichment for transcripts with known critical roles in decidualization. Importantly, one of the pathways highly enriched in our IPA was the WNT/β-catenin pathway, which includes several genes (*Wnt4*, *Wnt6*, *Wnt16*, *Fzd6*, *Foxa2*) found to be dysregulated in Hoxa11^+/−KO BMT^ as compared with WT^WT BMT^ and Hoxa11^+/−WT BMT^. The *Wnt* genes encode secreted glycoproteins that are homologous to the *Drosophila* segment polarity gene *wingless* (*wg*) and control essential developmental processes, such as embryonic patterning, cell growth, migration, and differentiation [68]. A subset of Wnt genes (*Wnt4*, *Wnt5a*, *Wnt6*, *Wnt7a*) are involved in female reproductive tract development and are critical for decidualization and implantation [51–53]. Interestingly, it was shown that *Wnt7a*-null mice have defective patterning of the uterus and absence of glands associated with loss of uterine Hoxa11 expression [69], suggesting that *Wnt7a* is upstream of Hoxa11. Moreover, mice with conditional KO of *Wnt7a* in the uterus using the Pgr^Cre^ mouse model have defective decidualization and implantation, which is associated with aberrant uterine gene expression during implantation, including increased *Wnt4* and decreased *Wnt16* and *Foxa2* [70]. Consistent with these studies, we observed decreased expression of *Wnt16*, *Fzd6*, and *Foxa2* in the Hoxa11^+/−KO BMT^ as compared with Hoxa11^+/−WT BMT^. Another signaling pathway with a critical role in decidualization that was significantly dysregulated in Hoxa11^+/−KO BMT^ but normalized by BMT from WT donors is prolactin signaling. Several members of the prolactin superfamily with known roles in decidualization (*Prl3c1*, *Prl6a1*, *Prl8a2*) [40,49,50] were increased in Hoxa11^+/−KO BMT^ as compared with Hoxa11^+/−WT BMT^ and WT^WT BMT^ mice. It is plausible that Hoxa11 dysregulation in Hoxa11^+/−KO BMT^ and associated decidual dysfunction leads to compensatory increase in these decidualization factors. Taken together, our findings suggest that BMDCs transform the uterine implantation transcriptome of Hoxa11^+/−^ mice in favor of normal decidualization and are crucial for pregnancy maintenance.

Lineage tracing of nonhematopoietic BM populations was not performed in this study, as Hoxa11 expression in the BM is restricted to mesenchymal stem/stromal cell progenitors and it is not expressed in hematopoietic cells [25]. Thus, Hoxa11 expression in transplanted BM cells should be restricted to the nonhematopoietic BM subpopulation. Our flow cytometry results in Hoxa11^+/−^ mice with GFP knock-in are consistent with this, showing specific expression of Hoxa11 in nonhematopoietic BM and uterine stromal cells and absence of Hoxa11 expression in hematopoietic BM cells. Moreover, our Hoxa11-GFP BMT model demonstrated that all Hoxa11^+^GFP^+^ BMDCs recruited to the pregnant uterus were nonhematopoietic. In contrast, Hoxa11^+^GFP^+^ cells were not found in the nonpregnant uterus. Taken together, these data suggest that the positive effects of BMT on uterine stromal expansion and decidualization in Hoxa11 KO mice, and pregnancy loss prevention in Hoxa11^+/−^ mice in our study, are induced by the nonhematopoietic BMDC Hoxa11-expressing subpopulation rather than the hematopoietic BMDCs. Moreover, recruitment of BMDCs to the uterus, total leukocyte numbers, as well as immune cell subpopulations in the uterus did not appear to be altered in nonpregnant Hoxa11^−/−^ or pregnant Hoxa11^+/−^ mice, further suggesting that hematopoietic and/or immune dysregulation is unlikely to explain the phenotypes in these mice.

While this study does not define all the BM cell subsets that give rise to DSC population in pregnancy, our study provides several lines of evidence in support of BM-MSC as a BM origin of these nonhematopoietic cells: GFP^+^ BM-MSC populations are reconstituted in the BM following BMT in our model; these BM-MSCs are found to be mobilized to the circulation in response to pregnancy; BM-MSCs are able differentiate into stromal decidual cells in vitro; and Hoxa11^+^GFP^+^ nonhematopoietic BMDCs are found in the pregnant uterus. Another study similarly showed that human BM-MSCs can differentiate in vitro into prolactin-producing stromal cells [71]. In this study, we used established BM culture techniques for expansion and enrichment of MSCs, which would include mesenchymal stromal cell populations. The use of specific markers such as CD140a in the isolation procedure would have refined the MSC population. Previously, it was shown that endothelial progenitor cells (EPCs) are increased during human pregnancy [72] and that EPCs directly contribute to ECs within decidual vasculature by vasculogenesis in mouse pregnancy [73]. Other studies in mice and rats showed that hypoxia, ischemia, and liver injury induced the mobilization of MSCs to peripheral blood [38,74,75], suggesting that systemic signals trigger the release of MSCs from the BM. Interestingly, hypoxia- and ischemia-induced mobilization appears to be specific for MSCs because total circulating HSCs were not significantly increased [38,74]. Similarly, pregnancy-induced mobilization in our study was specific for MSCs, as HSCs were not affected.

Previous studies have shown that factors driving the recruitment of BM-derived nonhematopoietic cells to the endometrium include uterine ischemia and injury [22,76], which increased engraftment of BMDCs to the stromal compartment by 2-fold, and estrogen, which increased the incorporation of BM-derived endothelial progenitors into uterine vasculature [77]. In contrast, exposure to cigarette smoke, which has been linked to infertility, decreases the recruitment of both stromal and epithelial BMDCs [78]. Our study shows that implantation and the developing pregnancy are very strong physiological stimuli for BMDC engraftment into the uterus (an approximately 4-fold increase on E9.5 compared with the nonpregnant state). As it was demonstrated that CXCL12 ligand and its CXCR4 receptor are important in mediating migration of BMDCs towards endometrial stromal cells in vitro [79], one may speculate that the CXCL12/CXCR4 axis plays a role in mediating homing of BMDCs to the decidua at the time of implantation and pregnancy. However, future studies are warranted to investigate the exact mechanism(s) responsible for BMDC recruitment to the uterus in response to pregnancy.

Recurrent pregnancy loss (RPL) in humans, defined as the loss of three or more consecutive pregnancies prior to 20 weeks of gestation, affects 1% to 2% of couples and in over 50% of cases its cause remains unexplained [80]. With the advent of in vitro fertilization (IVF), recurrent implantation failure (RIF), when embryos fail to implant after several IVF treatment attempts, is an increasingly recognized clinical condition [81]. The Hoxa11-deficient mice used herein model both of these clinical conditions; the Hoxa11^−/−^ mice have a more severe and earlier defect, leading to recurrent failure of implantation, while the Hoxa11^+/−^ mice have partial Hoxa11 expression characterized by apparently normal implantations followed by pregnancy losses. Hoxa11 expression has also been implicated in human implantation, and several studies have shown uterine Hoxa11 expression to be decreased in conditions associated with pregnancy failure, such as submucosal leiomyomas, endometriosis, and pregnancy loss [46,82–84]. Moreover, it was reported that women with RPL and obesity-associated reproductive failure have decreased endometrial clonogenic cell populations and accelerated stromal cell senescence, suggesting endometrial stem cell dysfunction [85,86]. Our findings that Hoxa11-expressing BMDCs promote changes in the uterine transcriptome leading to partial correction of decidualization defect in Hoxa11^−/−^ mice and full rescue of pregnancy loss in Hoxa11^+/−^ mice raise the possibility that BM stem cell dysfunction may be underlying RIF and/or RPL, and that stem cell therapy may prove to be a viable approach to treat these conditions.

In summary, our study provides evidence that BMDCs have a nonhematopoietic physiologic contribution to the decidual stroma and play an important role in implantation and pregnancy maintenance. Importantly, nonhematopoietic BMDCs have the ability to influence the decidual molecular milieu and overcome implantation defects. Although it remains to be established to what extent these findings in the mouse translate to the situation in humans, our data raise the prospect that BMDCs dysfunction may contribute to implantation failure or pregnancy loss in women.

## Materials and methods

### Ethics statement

Mice were maintained in the Animal Facility of Yale School of Medicine and were treated under an approved Yale University Institutional Animal Care and Use Committee protocol (#07113).

### Animal studies

Hoxa11 KO mice (B6.129-Hoxa11^tm1Dmwe^/J) were previously described and purchased from Jackson Laboratories (Bar Harbor, ME) (Stock no. 011036). C57BL/6J WT mice, transgenic ubiquitin-GFP mice C57BL/6-Tg(UBC-GFP)30Scha/J (Stock no. 004353), mTmG mice B6.129(Cg)-Gt(ROSA)26Sor^tm4(ACTB-tdTomato,-EGFP)Luo^/J (Stock no. 007676), and β-actin-Cre mice B6N.FVB-Tmem163^Tg(ACTB-cre)2Mrt^/CjDswJ (Stock no. 019099) were all purchased from Jackson Laboratories (Bar Harbor, ME).

### Submyeloablation and BMT based on 5-FU

Submyeloablation was performed using the 5-FU–based CTX-3+SCF protocol as previously described [24]. General toxicity of the treatment regimen was monitored by measuring weights of the mice and assessing their well-being daily. For the Hoxa11 functional experiments, BMT was performed from either WT or Hoxa11^−/−^ male donor mice into WT, Hoxa11^+/−^, or Hoxa11^−/−^ females. BMTs were performed according to the procedure described previously [24]. Briefly, marrow cells were obtained from 6- to 10-week-old C57BL/6J male mice by flushing the marrow from femurs and tibias into cold sterile PBS, filtered through 70-μm mesh, and 20 × 10^6^ unfractionated BM cells were injected IV into 6- to 7-week-old female Bl/6 recipients on day 0 after conditioning with the BM regimen described above.

### Experimental models

For time course experiments to characterize BMDCs in pregnancy, BMT was performed from syngeneic male GFP mice into myeloablated C57BL/6J WT 6- to 7-week old female mice. For the cell fusion experiments, BMT was performed from mTmG male donor mice into either β-actin-Cre or WT 6- to 7-week-old female mice. Following a 3-week recovery period, BM engraftment was checked in peripheral blood, and only mice with >30% BMDC chimerism were used for subsequent experiments. Female mice were bred with fertility-proven C57BL/6J male mice and checked for vaginal plugs at 7 AM daily. The morning of vaginal plug was considered E0.5. Successfully bred female mice were killed at various gestational time points, including E5.5, E9.5, E13.5, and E17.5. In addition, virgin mice that underwent BMT from GFP donors served as the nonpregnant group. For each gestational time point, pregnant mice that did not undergo BMT and were injected with PBS instead of BM cells served as controls. The engraftment of GFP-positive BMDCs and their characterization in the uterus and uterine implantation sites was performed by fluorescent camera, flow cytometry, single-cell RNAseq, and immunohistochemistry/fluorescence.

For the Hoxa11 functional experiments, BMT was performed from either WT or Hoxa11^−/−^ male donors into myeloablated WT, Hoxa11^+/−^, or Hoxa11^−/−^ 6- to 7-week-old female mice. Following a 3-week recovery period, different experiments were performed. For the litter size experiment, mice were harem bred with fertility-proven WT males for 1 month and monitored for weight daily. A weight increase of 4 g was considered a sign of pregnancy, at which point the pregnant dam was separated from the male and housed singly until delivery. Following delivery, the litter size and pup weights were recorded and time to delivery was calculated. For the implantation experiment (E5.5), transplanted mice were bred and checked for vaginal plugs daily as described above. Successfully bred females were killed on E5.5 and the number of implantation sites in the uterus was counted. Then, the implantation sites were extracted for histology, immunohistochemistry/fluorescence, protein analysis, and RNA expression analysis. For the resorption experiment, plug positive female mice were killed on E12.5, and the uterus was inspected for presence of resorptions. Mean resorption rate per pregnancy, no. of viable implantations per mouse, and percentage of dams with resorptions were calculated. To assess the effects of BMT on the Hoxa11^−/−^ uterus in the nonpregnant state, virgin Hoxa11^−/−WT BMT^ and Hoxa11^−/−KO BMT^ mice were killed 4 weeks following BMT at different estrus cycle phases, and the uterus was weighed and extracted for histological analysis and immunohistochemistry.

For the Hoxa11 BMDCs recruitment experiments, BMT was performed from mTmG mice into WT, Hoxa11^+/−^, or Hoxa11^−/−^ females. For the Hoxa11-GFP biodistribution experiments, BMT was performed from Hoxa11-GFP mice into WT mice. Following a 3-week recovery period, transplanted mice were bred and checked for vaginal plugs daily as described above, or killed as virgins. BM, spleen, implantation site, or nonpregnant uterus was extracted and processed as described below.

### Fluorescent imaging of organs

After organs were extracted, they were imaged with Carestream In-Vivo MS Fx Pro (Carestream Health, USA) to capture X-ray and corresponding GFP fluorescent images.

### Processing of uterus, BM, spleen, and blood samples and flow cytometry analysis

Uterine implantation sites were extracted and fetal/placental parts were removed from the uterus. The uterine tissues were finely minced and subsequently digested with a solution of Hanks' balanced salt solution (Life TechnologiesSan Francisco, CA) containing HEPES (25 mM), collagenase B (1 mg/mL; Roche Diagnostics, Indianapolis, IN), and deoxyribonuclease I (0.1 mg/mL; Sigma-Aldrich, St. Louis, MO) for 30 to 45 minutes at 37°C. The cell suspension was filtered through a 70-μm filter and centrifuged at 2,000 rpm for 8 minutes at 4°C. The cell pellet was washed with PBS by centrifugation at 2,000 rpm for 5 minutes at 4°C and then resuspended in PBS with 2% FBS (FACS buffer). Blood was obtained by the retro-orbital method and collected in an EDTA-coated tube, followed by transfer to 1 mL PBS and centrifugation at 1,500 rpm for 5 minutes at 4°C, followed by RBC lysis for 10 minutes at room temperature and resuspension in FACS buffer. Cells were then washed once in FACS buffer. BM cells were flushed from femur and tibia in PBS, centrifuged at 1,500 rpm for 5 minutes at 4°C, followed by RBC lysis and further processing as described above for blood. Spleen was crushed and cells were resuspended in PBS, centrifuged at 1,500 rpm for 5 minutes at 4°C, followed by RBC lysis and further processing as described above for blood. Total extracted cells per mouse were counted using a hemocytometer. Cell suspensions were then incubated with mouse Trustain FcX PLUS anti CD16/32 (Biolegend, San Diego, CA) blocking for 10 minutes, followed by incubation with the appropriate antibodies at room temperature for 30 minutes. The cells were washed with FACS buffer twice, resuspended in PBS, and then analyzed on a fluorescence-activated cell sorting Beckman Coulter MoFlo machine (Beckman Coulter). Gates were applied to forward-scatter/side-scatter dot plots to exclude nonviable cells and cell debris. Appropriate unstained and antibody IgG isotype controls were used for setting compensation and determining gates. Data were analyzed using the software FlowJo V10 (FlowJo). Antibodies used in flow cytometry analysis are listed in S1 Table.

### BM MSC culture, trilineage differentiation, and in vitro decidualization

BM cells were extracted from GFP BM-transplanted mice after flushing from the femur and tibia into DMEM-F12 medium. Cells were filtered through a 70-um filter and centrifuged at 2,000 rpm for 8 minutes at 4°C, then resuspended in MSC expansion medium containing MesenCult Basal Medium, MesenCult 10X Supplement, MesenPure, L-Glutamine, 1% penicillin/streptomycin antibiotic (MesenCult Expansion Kit (Mouse), Stem Cell Technologies, Cambridge, MA). Cells were counted and 5 × 10^7^ cells were plated in a T75 flask in 15 mL MesenCult medium. They were cultured at 37°C under 5% CO_2_. After 1 week, cells were passaged and maintained until the second passage (P2). P2 cells were used for subsequent flow cytometry characterization, MSC trilineage differentiation, and decidualization experiments.

MSC trilineage differentiation into adipogenic, osteogenic, and chondrogenic lineage was performed according to the manufacturer’s instructions (R&D systems, Minneapolis, MN) using P2 cultured MSCs. For adipogenic differentiation, cells were plated at a concentration of 4 × 10^4^ cells per well in a 12-well plate. For osteogenic differentiation, cells were plated at a concentration of 2 × 10^4^ cells per well in a 12-well plate. After 14 days in adipogenic medium and 18 days in chondrogenic medium, immunocytochemistry was performed according to the manufacturer’s instructions (R&D systems, Minneapolis, MN). For chondrogenic differentiation, 2 × 10^6^ cells were centrifuged, washed once, and then the pellet was resuspended in 0.5 mL chondrogenic differentiation medium in a 15-mL falcon tube followed by centrifugation and incubation upright at 37°C and 5% CO_2_. Fresh Chondrogenic Differentiation Medium was replaced every 2 days, with caution not to disrupt the pellet. Another pellet was similarly processed but incubated in chondrogenic basal medium as negative control. After 21 days in culture, the pellets were fixed in 4% paraformaldehyde and embedded in paraffin. Five-micrometer tissue sections were mounted on slides, followed by immunocytochemistry as per the manufacturer’s instructions (R&D systems, Minneapolis, MN). Images were captured using a fluorescent microscope (Axio Observer, Carl Zeiss). Trilineage differentiation experiments were performed in triplicate and repeated twice.

For decidualization experiments, P2 cultured MSCs were plated at concentration of 1 × 10^5^ cells per well in a 12-well plate in αMEM medium + 10% FBS. After reaching 90% confluence, cells were serum starved overnight, followed by treatment with basal medium (αMEM_,_ 2% FBS) containing either 0.5 mM 8-bromoadenosine cAMP (Sigma Aldrich, St. Louis, MO), 1 μM 17-MPA (Sigma Aldrich, St. Louis, MO), combination of 0.5 mM cAMP + 1 μM MPA, or control basal medium only. Medium was replaced every 3 days and cells were cultured for a total of 14 days. As positive control for decidualization, mouse uterine stromal cells were isolated by mild collagenase digestion and cultured in DMEM/F12 medium supplemented with 10% FBS and penicillin/streptomycin (100 μg/mL) as previously described [79]. They were plated at concentration of 1 × 10^6^ cells per well in a 6-well plate and cultured with a combination of cAMP and MPA or control medium for a total of 14 days. BM MSC cells and uterine cells were analyzed for Prl8a2 mRNA on day 3, day 8, and day 14. Alternatively, cells were stained for F-actin after 9 days to visualize decidual morphological changes. All decidualization experiments were performed in triplicate and repeated three times.

### F-actin staining

F-actin staining of BM MSC cells and uterine cells was performed after 9 days in culture with different decidualization treatments. Cells were washed three times with PBS, fixed in 4% PFA, and then blocked with 3% BSA. This was followed by staining with rhodamine phalloidin (Molecular Probes, San Francisco, CA) according to the manufacturer’s instructions. Mounting was done under coverslips using Vectashield fluorescent mounting media with DAPI (Vector Laboratories, Burlingame, CA). Images were captured using a fluorescent microscope (Axio Observer, Carl Zeiss).

### Western blot

Proteins extracted (25 μg) from E.D 5.5 pregnant mouse uteri were separated on 4%–15% TGX gel (Mini-PROTEAN TGX, Biorad) and were transferred onto a PVDF membrane (Biorad, Wallingford, CT), followed by blocking with 5% milk. Membranes were probed with HOXA11 antibody (rabbit, Novus biologicals, Littleton, CO; NBP1-80228) and GAPDH antibody (rabbit, Cell Signaling, Danvers, MA; 2118S) followed by incubation with HRP linked secondary antibody (goat anti-rabbit, Cell Signaling; 7074S) in 5% milk. Membranes were incubated in chemiluminescence substrate and exposed to film.

### Histology and immunohistochemistry

Uterine tissues were fixed in 4% paraformaldehyde and embedded in paraffin. Five-micrometer tissue sections were mounted on slides, followed by deparaffinization and rehydration. Slides were then boiled in sodium citrate (pH 6.0) for antigen retrieval. Sections were blocked with either 5% goat or donkey serum followed by incubating with primary antibody overnight at 4°C. Sections were then incubated with biotinylated secondary antibody (1:200; Vector Laboratories, Burlingame, CA) or fluorescent secondary antibody for 1 hour. For immunohistochemistry, detection was performed using ABC Vectorstain Elite reagents with DAB plus H2O2 (Vector Laboratories). Tissue sections were counterstained with hematoxylin (Sigma-Aldrich, St. Louis, MO). Images of stained sections were obtained using an Olympus BX-51 microscope (Olympus). For immunofluorescence and colocalization studies, Alexa Fluor 568-conjugated donkey anti-goat, Alexa Fluor 568-conjugated donkey anti-rabbit, Alexa Fluor 488-conjugated donkey anti-rabbit, and/or Alexa Fluor 488-conjugated donkey anti-rat (Life Technologies, San Francisco, CA) was used, as appropriate, followed by mounting under coverslips using Vectashield fluorescent mounting media with DAPI (Vector Laboratories). Immunoreactions with amplification but without primary and/or secondary antibodies were performed as controls. Images were captured using laser scanning confocal microscope (LSM 710, Carl Zeiss) and analyzed using ZEN software (Carl Zeiss). Primary and secondary antibodies and their respective concentrations used are listed in S1 Table.

### Image quantification and analysis

For quantitation of proliferating GFP-positive and GFP-negative cells in the endometrial stromal or decidual compartments, 12 high-power confocal microscopy fields (4 high-power fields [HPFs] from each of 3 uterine sections per animal) were assessed for each gestational time point. The total number of DAPI-positive cell nuclei and GFP-positive cells were counted in each HPF, and the number of GFP^+^/PCNA^+^ and GFP^−^/PCNA^+^ was then counted and expressed as a percentage of the total GFP^+^ or GFP^−^ cells counted per animal, respectively. At least 1,000 cells were counted per animal. Quantitation of DPRP^+^/GFP-positive and DPRP^+^/GFP-negative cells and quantitation of PR^+^/GFP-positive and PR^+^/GFP-negative cells in the endometrial stromal or decidual compartments was performed following the same methodology as described above. For quantification of PR, PCNA, HoxA11 IHC staining in implantation sites, 12 high-power microscopy fields (4 HPFs from each of 3 uterine sections per animal) were assessed for each type of staining. For quantification of percent PR-positive and percent PCNA-positive cells, the total number of cells that stained positive for PR or PCNA in implantation sites was divided by the total number of counted cells. For quantification of HoxA11 staining, the percentage of cells positive for HoxA11 stain in the PDZ was calculated. For quantification of uterine stromal areas and stromal vessel luminal area, hematoxylin–eosin (HE) and CD31 stained photomicrographs of KO mice were analyzed using ImageJ Image analysis software (Rasband, W.S., ImageJ, MD). Results were averaged and expressed as total stromal area (μm^2^) or stromal area as a percentage of total endometrial area (%). Mean vessel luminal area was calculated and expressed as mean vessel area/HPF.

### RNA extraction

Total RNA was extracted from mice uterine tissues by homogenizing the tissues in 1 mL TRIzol (Life Technologies, San Francisco, CA) followed by the addition of 200 uL chloroform to the lysate for phase separation by centrifugation at 13,500 rpm for 15 minutes. RNA in the aqueous phase was then precipitated by the addition of 500 μL isopropanol followed by centrifugation at 13,500 rpm for 10 minutes. The RNA pellet was then washed twice with 1 mL 75% ethanol by centrifugation at 10,500 rpm for 5 minutes. The RNA pellet was allowed to dry and then re-dissolved in nuclease free water. Total RNA was purified via RNeasy spin columns (QIAGEN, Germantown, MD) followed by treatment with Dnase using the TURBO DNA-free kit (Life Technologies). For extraction of RNA from BM and uterine cell cultures, total RNA was extracted by disrupting the cells with 300 uL RLT buffer having β-mercaptoethanol, followed by processing in RNeasy spin columns (QIAGEN) as per the manufacturer’s protocol. This was followed by treatment with Dnase using the TURBO DNA-free kit (Life Technologies).

### Real-time RT-PCR

For each sample, 500 ng of total RNA was reverse transcribed using an iScript cDNA Synthesis Kit (Bio-Rad, Wallingford, CT) according to the manufacturer’s instructions. qPCR was performed using iQ SYBR Green Supermix (Bio-Rad) on a Bio-Rad CFX96 thermocycler using the gene-specific primer pairs listed in S2 Table. Gene expression was analyzed on duplicate samples and the Ct values were normalized to the GAPDH housekeeping gene.

### RNA-seq library preparation and sequencing

For RNA-seq from uterine tissues of implantation sites, total RNA was isolated by TRIzol extraction as described above. RNA concentration was determined via a NanoDrop 2000 (Thermo Fisher Scientific, Waltham, MA). RNA integrity analysis, library preparation, and sequencing were performed by the Yale Center for Genomic Analysis. RNA integrity was measured using an Agilent 2000 Bioanalyzer utilizing the Agilent RNA 6000 Pico Chip (Agilent, Santa Clara, CA) per the manufacturer’s specifications. Library preparation was performed with the Illumina TrueSeq Library Preparation Kit (Illumina, San Diego, CA) per the manufacturer’s specifications. Following first-strand synthesis with random primers, second-strand synthesis was performed with dUTP for generating strand-specific sequencing libraries. Libraries were sequenced on an Illumina HiSeq2500 with parameters set for high output, single-end chemistry, and 76-bp sequencing. Samples were multiplexed to six samples per lane, yielding an average of 37 million reads per sample.

### RNA-seq sequence alignment, quantification, imaging, and analysis

For each read, we trimmed the first 6 nucleotides and the last nucleotides at the point at which the Phred score of an examined base fell below 20 using in-house scripts. If, after trimming, the read was shorter than 45 bp, the whole read was discarded. Trimmed reads were mapped to the mouse reference genome (mm10) with a known transcriptome index (UCSC Known Gene annotation) with Tophat v2.1.1 [87] using the very-sensitive preset, first-strand library type and providing the corresponding gene model annotation. In these results, only the reads that mapped to a single genomic unique location, with a maximum of two mismatches in the anchor region of the spliced alignment, were reported. The default settings for all other Tophat options were used. The program Cufflinks v2.2.1 [88] was used to convert aligned reads generated from Tophat into relative expression values for each gene represented as FPKM (fragments per kilobase of exon per million mapped reads). Cuffdiff was used to obtain differential gene expression between the experimental groups using first-strand library type, providing gene model annotation and the genome sequence file for detection and correction of sequence-specific bias that can be caused by random hexamer during the process of library preparation.

Data were analyzed through the use of QIAGEN’s IPA (QIAGEN Redwood City, www.qiagen.com/ingenuity) and the KEGG pathway. IPA analysis was performed with default settings and threshold values of *q*-value ≤ 0.05.

### Tissue processing for scRNA-seq, single-cell capture, library preparation, and sequencing

We applied scRNA-seq using droplet microfluidics (10x Chromium) on a single-cell suspension dissociated from E9.5 uterine implantation site following removal of embryo/placental parts. The uterine decidual tissues were finely minced and subsequently digested with a solution of Hanks' balanced salt solution (Life Technologies) containing HEPES (25 mM), collagenase B (1 mg/mL; Roche Diagnostics), and deoxyribonuclease I (0.1 mg/mL; Sigma-Aldrich) for 30 minutes at 37°C. Thereafter, the cell suspension was filtered sequentially through a 70-μm filter, then through a 40-μm filter, and centrifuged at 2,000 rpm for 8 minutes at 4°C. The cell pellet was washed with PBS by centrifugation at 2,000 rpm for 5 minutes at 4°C and then resuspended in PBS with 0.1% FBS. Cell viability (>70% cells alive) and concentration were assessed by the Countess II Cell Counter (Life Technologies) to ensure the quality of cells. Nano-sized droplets that each contains a single cell with the bar-coded gel bead (GEMs) were generated using the Chromium controller (10x Genomics). The libraries were then created with Single Cell 3′ Library kit V2 according to the manufacturer’s protocol. Reverse transcription was performed with polyT primers containing cell-specific bar codes, unique molecular identifiers (UMIs), and adaptor sequences. All 10x libraries were sequenced in an Illumina HISeq 4000 instrument. We used the 10x Genomics Cell Ranger software v2.0.0 to align to the mm10 and its corresponding gene annotation, de-duplicate, filter bar codes, and quantify genes.

### scRNA-seq data processing and UMAP visualization

For graph-based clustering and differential gene expression analysis, we used Seurat 3.0 workflow [89]. In Seurat, an initial filter was applied to select only the cells that had a minimum of 200 unique transcripts; and to select only those genes that were expressed in at least 3 cells. For normalization and variance stabilization, we used the R package sctransform (Hafemeister and Satija, bioRxiv 2019 https://doi.org/10.1101/576827) which has a direct interface to Seurat toolkit. During normalization with sctransform, we also included in the model the mitochondrial mapping percentage as an unwanted source of variation. Dimensionality reduction and graph-based clustering was performed on the transformed data with PCA and UMAP algorithm [90]. The expression of established lineage marker genes was used to assign cell types.

### Statistical analysis

Data were assessed for normal distribution with a Shapiro-Wilk normality test using GraphPad Prism 6 software (GraphPad Software, La Jolla, CA). Normally distributed data were analyzed using the Student unpaired two-tailed *t* test for the comparison of two groups, and one-way ANOVA with Tukey multiple comparison test for multiple group comparison. If data were not normally distributed, or if distribution could not be determined due to small sample size, data were analyzed using a Mann-Whitney U test. For time to delivery Meier-Kaplan curves, differences between curves were evaluated by applying the log-rank test (Kaplan-Meier log-rank value, *p*-value). *p* < 0.05 was considered statistically significant.

## Supporting information

S1 Fig (A) A schematic of the 5-FU–based submyeloablation protocol used for non-gonadotoxic BM transplantation. (B) Imaging using a fluorescent camera following dissection to separate the uterus (U), placenta (P), and embryo (E) on E9.5, demonstrating the engraftment of GFP-positive BMDCs in uterus. Right panel and middle panel are GFP fluorescence and X-ray images, respectively. BM, bone marrow; BMDC, BM-derived cell; E, embryo; GFP, green fluorescent protein P, placenta; U, uterus; 5-FU, 5-fluorouracil.
(TIF)

S2 Fig BM MSCs are reconstituted following 5-FU–based BMT. (A) Multicolor flow cytometry analysis of BM cells extracted from mice transplanted with BM from GFP donors following 5-FU submyeloablation. Cells were gated on GFP^+^ or GFP^−^ followed by gating on Sca1^+^ and lin^−^ to identify MSCs (Sca1^+^/CD45^−^/lin^−^) or HSCs (Sca1^+^/CD45^+^/lin^−^), *n* = 4. (B-F) Cultured BM cells from mice transplanted with BM from GFP donors following 5-FU submyeloablation, *n* = 4. Extracted BM cells were cultured, passaged, and P-2 cells consisted of adherent mixed GFP^+^ (green) and GFP^−^ cells (C). They were analyzed by multicolor flow cytometry (B). Cells were gated on GFP^+^ or GFP^−^ followed by gating on CD45^−^, CD29^+^, Sca1^+^, and CD44^+^ to identify cultured MSCs. (D-F) Fluorescent images of trilineage differentiation of P-2 cultured BM cells grown in adipogenic media (D), osteogenic media (E) or chondrogenic media (F). GFP+ cells are shown in green. FABP4 (D), osteopontin (E), or collagen II (F) are shown in red. Nuclei are stained with DAPI (blue). The bottom row for each panel is a higher magnification of the area in the middle row enclosed by a rectangle. (G-I) Cultured P-2 BM cells extracted from 5-FU–transplanted mice were serum starved for 24 hours followed by culturing with either 17-MPA, 8-bromoadenosine-3′,5′-cAMP (cAMP), MPA+cAMP, or control medium for 14 days. Primary P-2 mouse uterine stromal cells served as positive control for decidualization. (G) Representative fluorescent images of cultured BM cells or uterine stromal cells after 14 days in culture showing F-actin filaments stained with phalloidin (red) and nuclei with DAPI (blue) demonstrating characteristic decidual morphologic changes most pronounced following cAMP and MPA+cAMP treatments. (H) Decidual Prl8a2 mRNA expression in BM cells on day 3, day 8, and day 14 of culture following MPA, cAMP, MPA+cAMP relative to control treatments. (I) Prl8a2 mRNA expression in uterine stromal cells following MPA+cAMP on day 3, day 8, and day 14. Values shown are expression levels relative to day 3. Results shown are the average of three independent experiments carried out in duplicates. Bar graphs represent mean ± SEM. *p < 0.01. **p < 0.05. Underlying data are available in S1 Data. BM, bone marrow; BMT, BM transplant; cAMP, 3′,5′-cyclic AMP;; FABP4, Fatty acid binding protein 4; GFP,green fluorescent protein; HSC, hematopoietic stem cell; lin, lineage; MPA, medroxyprogesterone acetate; MSC, mesenchymal stem cell; Prl8a2, prolactin-related protein; Sca1, stem cell antigen 1; 5-FU, 5-fluorouracil.
(TIF)

S3 Fig Flow cytometry profile of BM-derived (GFP^+^) peripheral blood cells in 5-FU myeloablated nonpregnant mice. (A) Multicolor flow cytometry analysis of peripheral blood cells extracted from mice transplanted with BM from GFP donors following 5-FU submyeloablation. Cells were gated on GFP^+^ followed by gating on Sca1^+^ and lin^−^ to identify MSCs (Sca1^+^/CD45^−^/lin^−^) or HSCs (Sca1^+^/CD45^+^/lin^−^). Percentages shown are of total live GFP^+^ cells, *n* = 6. (B) Histograms represent counts of GFP^+^ cells from peripheral blood that were stained with the indicated antibodies (blue line) and respective isotype controls (filled) (*n* = 4). (C) Quantification of percentage of circulating BM-derived (GFP^+^) cells expressing the various cell surface markers shown in (A) (*n* = 4). Bar graphs represent mean ± SEM. Underlying data are available in S1 Data. BM, bone marrow; GFP, green fluorescent protein; HSC, hematopoietic stem cell; lin, lineage; MSC, mesenchymal stem cell; Sca1, stem cell antigen 1; 5-FU, 5-fluorouracil.
(TIF)

S4 Fig Transverse histological section of E 9.5 uterus from mouse transplanted with BM from GFP donor showing the localization of BMDCs stained with anti-GFP antibody (brown). In the middle is the low-magnification image showing the mesometrial and antimesometrial sides of the implantation site. The mesometrial side is the side where the placenta, decidua basalis (DB), and the major blood vessels are located. The mesometrial lymphoid aggregate (MLAp) is a transient structure between the myometrial layers that surrounds the radial branches of the uterine artery. The antimesometrial side contains the rest of the maternal decidua in contact with the invading trophoblast. (A, B) Images of the placenta showing relative absence of GFP-positive cells on the fetal side. Red dashed line demarcates the giant cell (GC) layer. Maternal vascular spaces (black dash) have brown GFP-stained platelets (black arrows) and are interspersed between trophoblast cells and fetal vascular spaces (green dash). Red arrows point to nucleated red blood cells characteristic of fetal vascular spaces. (C, D) Images of the DB showing numerous GFP-positive BMDCs in the decidua. Red dashed line demarcates the GC layer. (E and F) Images of the outer part of the DB and MLAp showing numerous GFP-positive BMDCs. (G-L) Images of the antimesometrial side showing GFP-positive BMDCs in the antimesometrial decidua, where NK cells are not found. Red dashed line demarcates the GC layer. Red arrows point to some decidual cells. A star demarcates the new lumen. Scale bars, 100 μm. BM, bone marrow; BMDC, BM-derived cell; BV, blood vessel; DB, decidua basalis; GC, giant cell; GFP, green fluorescent protein MLAp, mesometrial lymphoid aggregate; NK, natural killer; SA, spiral artery; UC, umbilical cord.
(TIF)

S5 Fig Uterine sections from nonpregnant or E9.5 pregnant mice showing immunofluorescence co-staining with macrophage marker F4/80 (red) and GFP (green) antibodies in nonpregnant uterus and E9.5 implantation site. Sections were counterstained with DAPI showing nuclei (blue). The images on the right of each panel are higher magnification of the corresponding dashed areas. Scale bars, 50 μm. GFP, green fluorescent protein.
(TIF)

S6 Fig Single-cell RNA-seq analysis of E9.5 implantation site. (A) Expression data dot plots of known lineage markers of DSCs, immune cells, ECs, and FBs. (B) Clustering of immune cells and DSCs following UMAP-based visualization of expression differences for cells using established lineage markers and (C) the same plot showing eGFP expression distribution within the same clusters. (D) Expression data dot plots of known lineage markers of eGFP^+^ and eGFP^−^ cells within immune cell and DSC clusters. DSC, decidual stromal cell; EC, endothelial cell; eGFP, enhanced green fluorescent protein; FB, fibroblast; RNA-seq, RNA sequencing; UMAP, Uniform Manifold Approximation and Projection.
(TIF)

S7 Fig Single-cell RNA-seq analysis of the proliferation status of immune and nonhematopoietic cells in the E9.5 implantation site. (A and B) Feature plots showing Mki67 (A) and PCNA (B) proliferation marker expression distribution within the total immune cell and DSC clusters. (C) mRNA expression levels and percentage of single Mki67^+^ and PCNA^+^ cells within GFP^+^ immune cells and GFP^+^ DSCs identified by single-cell RNA-seq. Percentages shown are of cells positive for the respective marker in each group. DSC, decidual stromal cell; GFP, green fluorescent protein; NS, not significant; PCNA, proliferating cell nuclear antigen; RNA-seq, RNA sequencing.
(TIF)

S8 Fig BMDCs do not fuse with host decidual cells. mT/mG transgenic mice were used as BM donors in 5-FU–based BMT into β-actin-Cre (ACTB-Cre) mice expressing Cre ubiquitously. The BM transplants from mT/mG donor into WT mice served as negative controls. Top panel shows flow cytometry of peripheral blood cells from mT/mG transgenic control; mice co-expressing Cre recombinase transgene under β-actin-Cre promoter as positive controls for the efficiency of Cre-mediated conversion from mT to mG in this system; and mTmG BMT into WT (control) or β-actin-Cre mice. Bottom panel shows flow cytometry of dissociated uterine implantation site cells (E9.5) of the same groups. Numbers in each quadrant indicate mean percentage of cells. *n* = 3–4 mice per group. BM, bone marrow; BMDC, BM-derived cell; BMT, BM transplant; WT, wild-type; 5-FU, 5-fluorouracil.
(TIF)

S9 Fig Pregnancy induces mobilization of MSCs to peripheral blood. Multicolor flow cytometry was performed on peripheral blood and BM cells of nonpregnant and pregnant mice on E5.5 and E9.5. Cells were gated on Sca1^+^ and Lin^−^ to identify stem cell populations and further divided into MSCs (Sca1^+^/CD45^−^/Lin^−^) and HSCs (Sca1^+^/CD45^+^/Lin^−^). (A) Representative flow cytometry graphs and quantitation of MSCs and HSCs populations in peripheral blood of nonpregnant, and E5.5 and E9.5 pregnant mice. (B). Quantitation of BM MSCs and HSCs in nonpregnant and E9.5 pregnant mice. *n* = 3–7 per group. *p < 0.01 versus nonpregnant group. Values and bar graphs represent mean ± SEM. **p < 0.05 versus E5.5 group. Underlying data are available in S1 Data. BM, bone marrow; HSC, hematopoietic stem cell; Lin, lineage; MSC, mesenchymal stem cell; Sca1, stem cell antigen 1.
(TIF)

S10 Fig Hoxa11 expression is restricted to nonhematopoietic cells. Hoxa11^+/−^ heterozygous mice in which GFP is knocked in, instead of the Hoxa11 gene, were used to identify distribution of Hoxa11 expression. Cells from BM (tibia or femur), spleen, peripheral blood, or implantation site of E9.5 dams were stained with CD45 pan-hematopoietic marker and subjected to flow cytometry analysis (*n* = 4 mice). BM, bone marrow; GFP, green fluorescent protein; Hoxa11, Homeobox a11.
(TIF)

S11 Fig Multicolor flow cytometry analysis of Hoxa11-GFP expression in uteri of WT pregnant (E9.5) and nonpregnant mice transplanted with BM from Hoxa11^+/−^ GFP donors. (A) Live single cells of nonpregnant or pregnant (E9.5) uterus showing that BM-derived Hoxa11/GFP^+^ cells are found in the pregnant uterus and are nonhematopoietic (CD45^−^). (B) E9.5 uterine cells were gated according to expression of CD45 and Hoxa11-GFP, as indicated by the arrows. NK1.1 and CD11b were used to identify NK cells and myeloid cells, respectively. Numbers in each quadrant indicate mean percentage of cells. *N* = 3–4 mice per group. BM, bone marrow; GFP, green fluorescent protein; Hoxa11, Homeobox a11; NK, natural killer; WT, wild-type.
(TIF)

S12 Fig IPA showing significantly enriched pathways of the genes commonly differentially expressed in the comparisons of Hoxa11^+/−KO BMT^ versus Hoxa11^+/−WT BMT^, and Hoxa11^+/−KO BMT^ versus WT^WT BMT^. BMT, BM transplant; Hoxa11, Homeobox a11; IPA, Ingenuity Pathway Analysis; KO, knockout; WT, wild-type.
(PDF)

S13 Fig Recruitment of BMDCs and leukocyte numbers to the uterus are unaltered in Hoxa11^−/−^ and Hoxa11^+/−^ mice. (A-H) Hoxa11^−/−^, Hoxa11^+/−^, or WT control mice were transplanted with BM from membrane Tomato (mT) donors. (A and E) Flow cytometry analysis of nonpregnant uterine cells of Hoxa11^−/−^ or WT mice (A) and of E9.5 implantation cells of pregnant Hoxa11^+/−^ and WT mice (E). Cells were stained with CD45 to identify leukocytes, and BMDCs are identified as tdTomato^+^. Representative graphs are shown. (B, F) Percentage CD45^+^ leukocytes out of total nonpregnant uterine cells of Hoxa11^−/−^ and WT (B) or out of total pregnant implantation site cells of Hoxa11^+/−^ and WT (F) are shown. (C and G) Percentage tdTomato^+^ BMDCs out of total nonpregnant uterine cells of Hoxa11^−/−^ and WT (C) or out of total pregnant implantation site cells of Hoxa11^+/−^ and WT (G) are shown. (D and H) Total number of BM cells per one hind limb (femur and tibia) and total number of splenic cells in nonpregnant Hoxa11^−/−^ and WT (D) or pregnant E9.5 Hoxa11^+/−^ and WT mice (H). Bar graphs represent mean ± SEM. *n* = 3–6 mice per group. Underlying data are available in S1 Data. BM, bone marrow; BMDC, BM-derived cell; Hoxa11, Homeobox a11; WT, wild-type.
(TIF)

S14 Fig Immune subpopulations in E9.5 implantation site of pregnant Hoxa11^+/−^ and WT mice. Top panel shows multicolor flow cytometry gating strategy. Live cells were gated according to forward and side scatters to exclude dead cells and debris, followed by single-cell gating with FSC-A and FSC-H. Live leukocyte single cells were gated on CD45. NK1.1 and Ly6G were used to identify NK cells and granulocytes, respectively. CD45^+^ cells were then gated on NK1.1^−^Ly6G^−^ to identify T cells (CD3^+^) and macrophages (F4/80^+^). T cells (CD3^+^ NK1.1^−^Ly6G^−^ F4/80^−^) were further classified as CD4^+^CD25^+^ Treg cells or CD4^+^CD25^−^ cells. Representative flow cytometry graphs are shown for WT and Hoxa11^+/−^ pregnant mice. Bottom bar graphs represent the mean percentage ± SEM for the various immune subpopulations. *n* = 4 mice per group. Underlying data are available in S1 Data. FSC-A, forward scatter area; FSC-H, forward scatter height; Hoxa11, Homeobox a11; NK, natural killer; Treg, T regulatory; WT, wild-type.
(TIF)

S15 Fig HE stained uterine sections from different phases of the estrus cycle of Hoxa11^−/−^ mice that received WT or KO BMT. The endometrial area in each photomicrograph is demarcated by a black dashed line. Notice the expanded endometrial stromal area and presence of endometrial glands (gl) only in Hoxa11^−/−WT BMT^ mice. BMT, BM transplant; gl, endometrial gland; HE, hematoxylin–eosin; Hoxa11, Homeobox a11; KO, knockout; WT, wild-type.
(TIF)

S16 Fig Lif mRNA expression levels in the implantation site on E5.5 in Hoxa11^+/−WT BMT^, Hoxa11^+/−KO BMT^, and WT^WT BMT^. *p < 0.05. Underlying data are available in S1 Data. BMT, BM transplant; Hoxa11, Homeobox a11; KO, knockout; Lif, Leukemia inhibitory factor; WT, wild-type.
(TIF)

S1 Table Primary and secondary antibodies. (DOCX)

S2 Table Primers for quantitative reverse transcription PCR (qRT-PCR). (DOCX)

S1 Data Underlying data. (XLSX)

S2 Data Four hundred ninety-eight genes commonly differentially expressed in E5.5 implantation sites between the comparisons of hetero_KO versus WT_WT and hetero_KO versus hetero_WT. KO, knockout; WT, wild-type.
(XLSX)

## Abbreviations

BM: bone marrow
BMDC: BM-derived cell
BMT: BM transplant
cAMP: 3′,5′-cyclic AMP
DBA: Dolichos biflorus agglutinin
DEG: differentially expressed gene
DPRP: decidual prolactin-related protein
DSC: decidual stromal cell
E: embryonic day
EC: endothelial cell
EGFP: enhanced green fluorescent protein
EPC: endothelial progenitor cell
FB: fibroblast
FDR: false discovery rate
GFP: green fluorescent protein
GO: gene ontology
HE: hematoxylin–eosin
Hox: homeobox-containing
Hoxa11: Homeobox a11
HPF: high-power field
HSC: hematopoietic stem cell
PA: Ingenuity Pathway Analysis
IVF: in vitro fertilization
KEGG: Kyoto Encyclopedia of Genes and Genomes
KO: knockout
Lif: leukemia inhibitory factor
Lin: Lineage
mG: membrane EGFP
MGI: Mouse Genome Informatics
MPA: medroxyprogesterone acetate
MSC: mesenchymal stem cell
Msx1: MSH homeobox 1
mT: membrane tdTomato
NK: natural killer
PAS: Periodic Acid Schiff
PBS: phosphate-buffered saline
PCNA: proliferating cell nuclear antigen
PCP: planar cell polarity
PDZ: primary decidual zone
PR: progesterone receptor
RIF: recurrent implantation failure
RPL: recurrent pregnancy loss
Sca-1: stem cell antigen-1
scRNAseq: single-cell RNA sequencing
Treg: T regulatory
UMAP: Uniform Manifold Approximation and Projection
UMI: unique molecular identifier
VEGFR2: vascular endothelial growth factor receptor 2
*wg*: *wingless*
Wnt: Wingless/Integrated
WT: wild-type
5-FU: 5-fluorouracil
