# Supplementary figures and images for: Adult bone marrow progenitors become decidual cells and contribute to embryo implantation and pregnancy
Source: PLoS Biol. 2019 Sep 12;17(9):e3000421. doi: 10.1371/journal.pbio.3000421 (PMC6742226; doi:10.1371/journal.pbio.3000421)

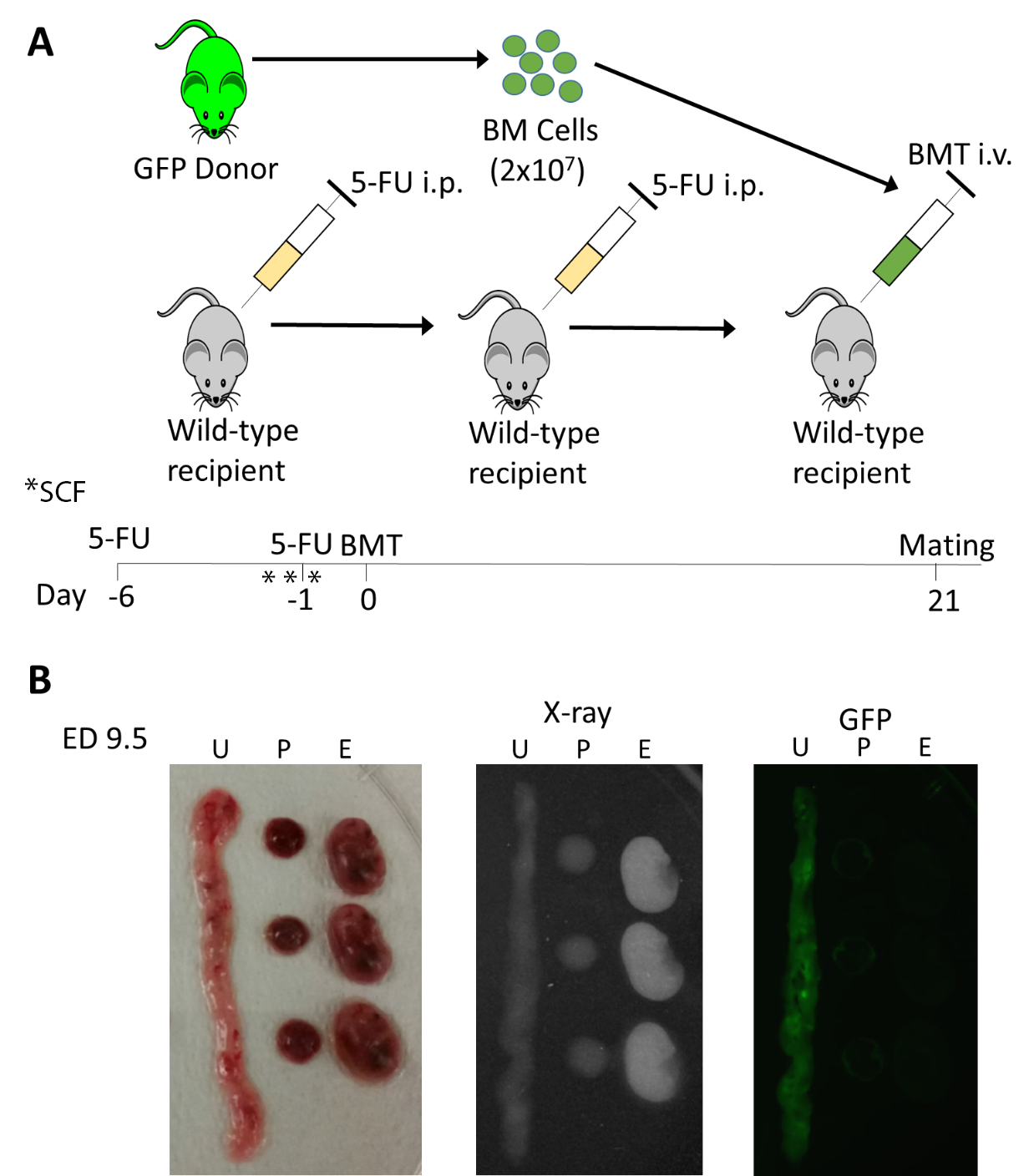

Supplement: S1 Fig — (A) A schematic of the 5-FU–based submyeloablation protocol used for non-gonadotoxic BM transplantation. (B) Imaging using a fluorescent camera following dissection to separate the uterus (U), placenta (P), and embryo (E) on E9.5, demonstrating the engraftment of GFP-positive BMDCs in uterus. Right panel and middle panel are GFP fluorescence and X-ray images, respectively. BM, bone marrow; BMDC, BM-derived cell; E, embryo; GFP, green fluorescent protein P, placenta; U, uterus; 5-FU, 5-fluorouracil. (TIF) [file pbio.3000421.s001.tif]

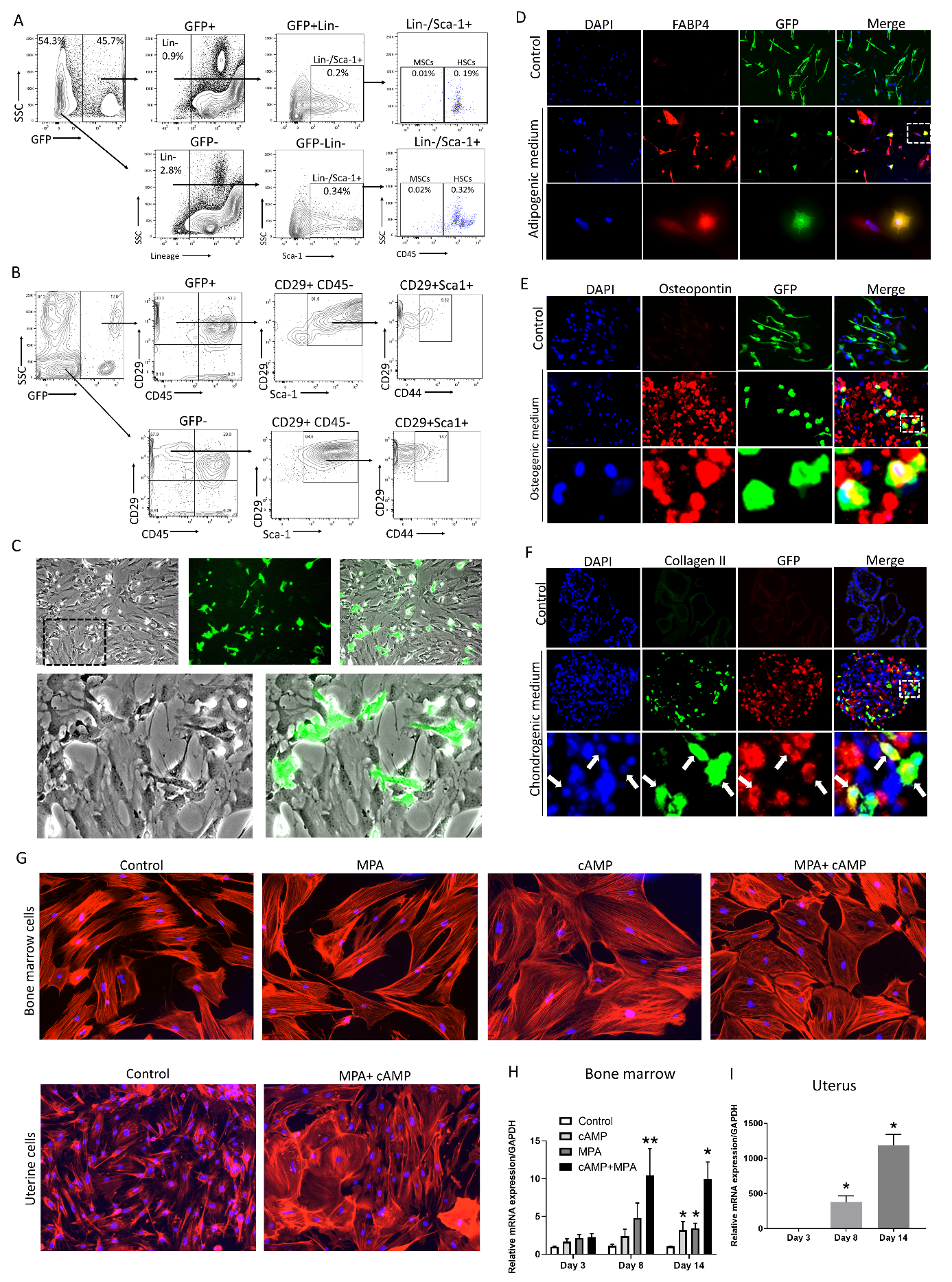

Supplement: S2 Fig — (A) Multicolor flow cytometry analysis of BM cells extracted from mice transplanted with BM from GFP donors following 5-FU submyeloablation. Cells were gated on GFP+ or GFP− followed by gating on Sca1+ and lin− to identify MSCs (Sca1+/CD45−/lin−) or HSCs (Sca1+/CD45+/lin−), n = 4. (B-F) Cultured BM cells from mice transplanted with BM from GFP donors following 5-FU submyeloablation, n = 4. Extracted BM cells were cultured, passaged, and P-2 cells consisted of adherent mixed GFP+ (green) and GFP− cells (C). They were analyzed by multicolor flow cytometry (B). Cells were gated on GFP+ or GFP− followed by gating on CD45−, CD29+, Sca1+, and CD44+ to identify cultured MSCs. (D-F) Fluorescent images of trilineage differentiation of P-2 cultured BM cells grown in adipogenic media (D), osteogenic media (E) or chondrogenic media (F). GFP+ cells are shown in green. FABP4 (D), osteopontin (E), or collagen II (F) are shown in red. Nuclei are stained with DAPI (blue). The bottom row for each panel is a higher magnification of the area in the middle row enclosed by a rectangle. (G-I) Cultured P-2 BM cells extracted from 5-FU–transplanted mice were serum starved for 24 hours followed by culturing with either 17-MPA, 8-bromoadenosine-3′,5′-cAMP (cAMP), MPA+cAMP, or control medium for 14 days. Primary P-2 mouse uterine stromal cells served as positive control for decidualization. (G) Representative fluorescent images of cultured BM cells or uterine stromal cells after 14 days in culture showing F-actin filaments stained with phalloidin (red) and nuclei with DAPI (blue) demonstrating characteristic decidual morphologic changes most pronounced following cAMP and MPA+cAMP treatments. (H) Decidual Prl8a2 mRNA expression in BM cells on day 3, day 8, and day 14 of culture following MPA, cAMP, MPA+cAMP relative to control treatments. (I) Prl8a2 mRNA expression in uterine stromal cells following MPA+cAMP on day 3, day 8, and day 14. Values shown are expression levels relative to day 3. Resu [file pbio.3000421.s002.tif]

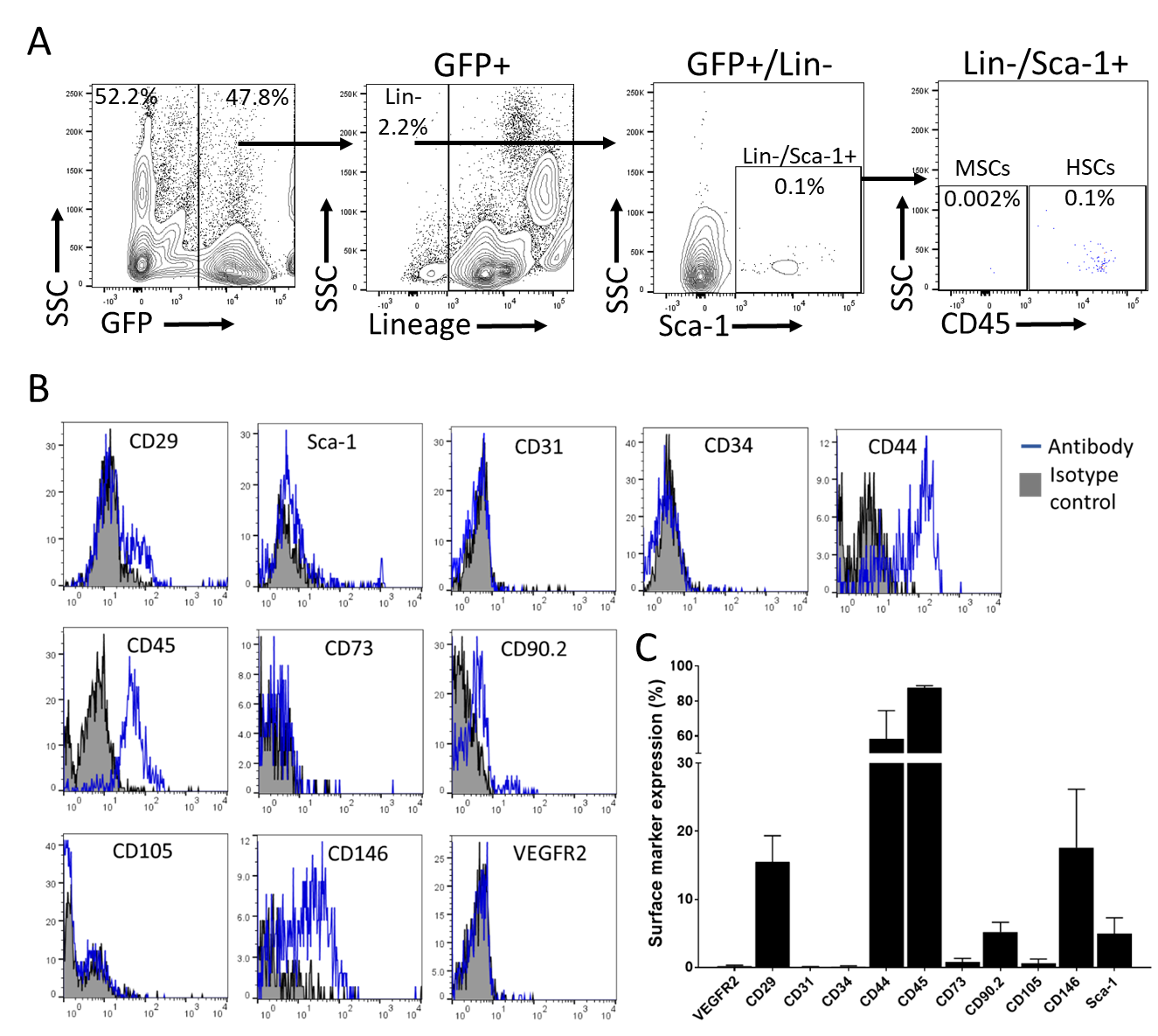

Supplement: S3 Fig — (A) Multicolor flow cytometry analysis of peripheral blood cells extracted from mice transplanted with BM from GFP donors following 5-FU submyeloablation. Cells were gated on GFP+ followed by gating on Sca1+ and lin− to identify MSCs (Sca1+/CD45−/lin−) or HSCs (Sca1+/CD45+/lin−). Percentages shown are of total live GFP+ cells, n = 6. (B) Histograms represent counts of GFP+ cells from peripheral blood that were stained with the indicated antibodies (blue line) and respective isotype controls (filled) (n = 4). (C) Quantification of percentage of circulating BM-derived (GFP+) cells expressing the various cell surface markers shown in (A) (n = 4). Bar graphs represent mean ± SEM. Underlying data are available in S1 Data. BM, bone marrow; GFP, green fluorescent protein; HSC, hematopoietic stem cell; lin, lineage; MSC, mesenchymal stem cell; Sca1, stem cell antigen 1; 5-FU, 5-fluorouracil. (TIF) [file pbio.3000421.s003.tif]

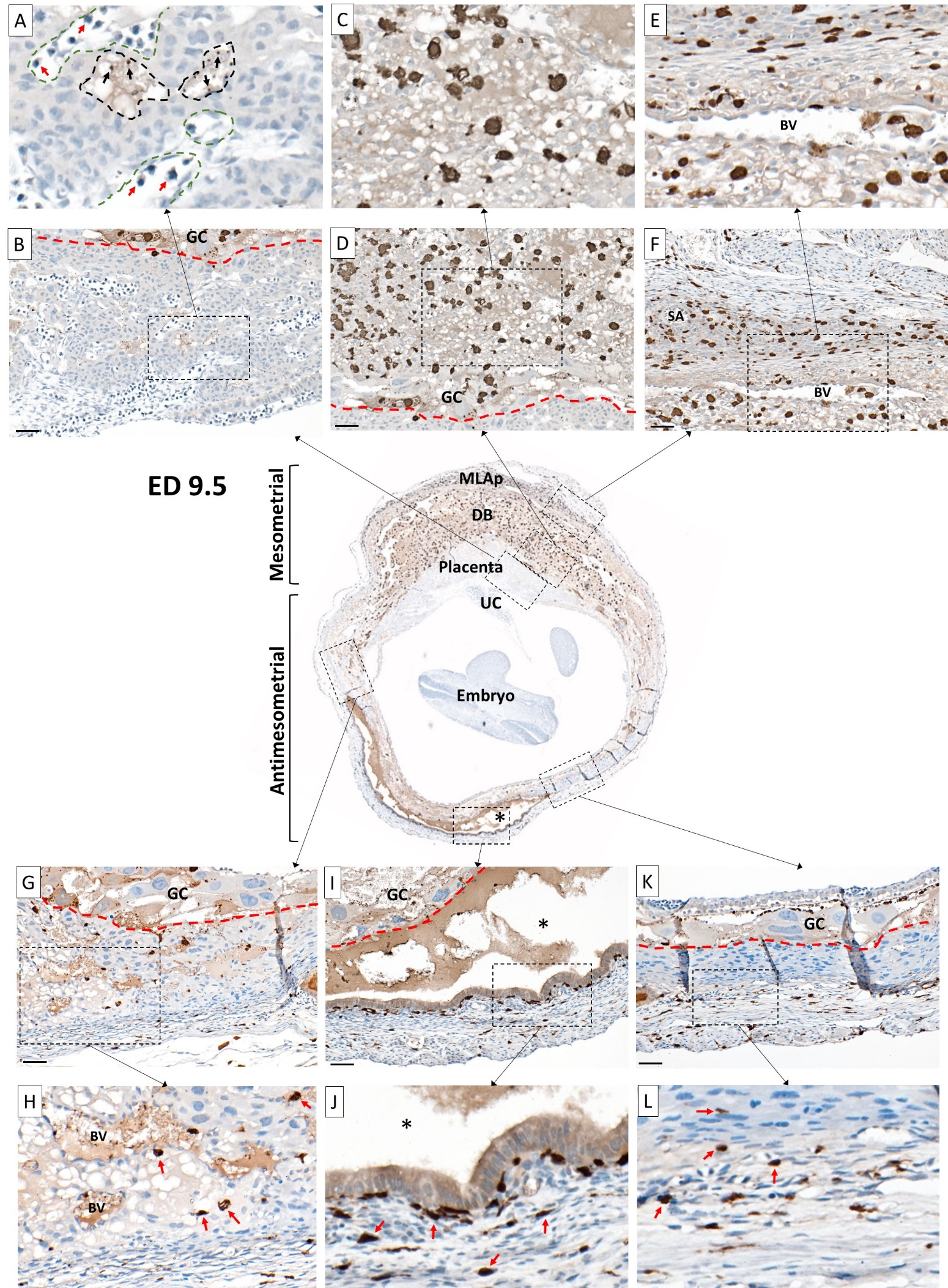

Supplement: S4 Fig — In the middle is the low-magnification image showing the mesometrial and antimesometrial sides of the implantation site. The mesometrial side is the side where the placenta, decidua basalis (DB), and the major blood vessels are located. The mesometrial lymphoid aggregate (MLAp) is a transient structure between the myometrial layers that surrounds the radial branches of the uterine artery. The antimesometrial side contains the rest of the maternal decidua in contact with the invading trophoblast. (A, B) Images of the placenta showing relative absence of GFP-positive cells on the fetal side. Red dashed line demarcates the giant cell (GC) layer. Maternal vascular spaces (black dash) have brown GFP-stained platelets (black arrows) and are interspersed between trophoblast cells and fetal vascular spaces (green dash). Red arrows point to nucleated red blood cells characteristic of fetal vascular spaces. (C, D) Images of the DB showing numerous GFP-positive BMDCs in the decidua. Red dashed line demarcates the GC layer. (E and F) Images of the outer part of the DB and MLAp showing numerous GFP-positive BMDCs. (G-L) Images of the antimesometrial side showing GFP-positive BMDCs in the antimesometrial decidua, where NK cells are not found. Red dashed line demarcates the GC layer. Red arrows point to some decidual cells. A star demarcates the new lumen. Scale bars, 100 μm. BM, bone marrow; BMDC, BM-derived cell; BV, blood vessel; DB, decidua basalis; GC, giant cell; GFP, green fluorescent protein MLAp, mesometrial lymphoid aggregate; NK, natural killer; SA, spiral artery; UC, umbilical cord. (TIF) [file pbio.3000421.s004.tif]

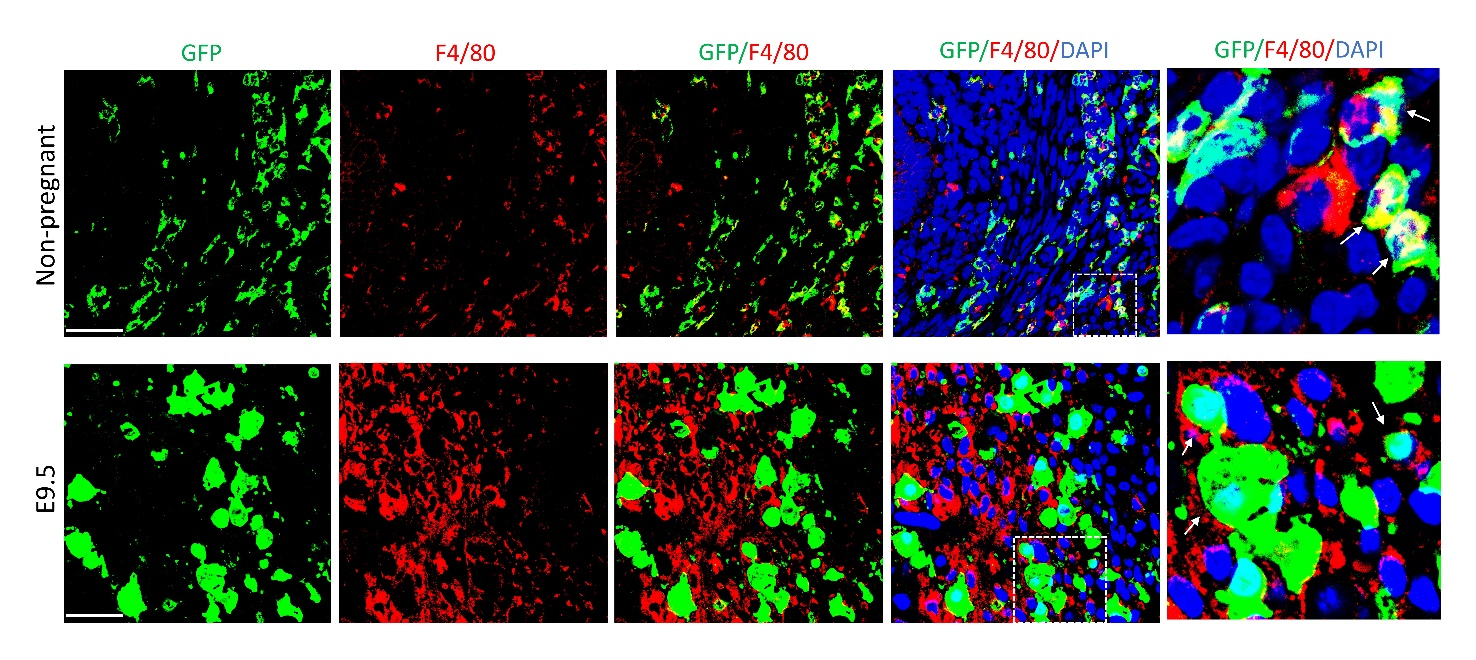

Supplement: S5 Fig — Sections were counterstained with DAPI showing nuclei (blue). The images on the right of each panel are higher magnification of the corresponding dashed areas. Scale bars, 50 μm. GFP, green fluorescent protein. (TIF) [file pbio.3000421.s005.tif]

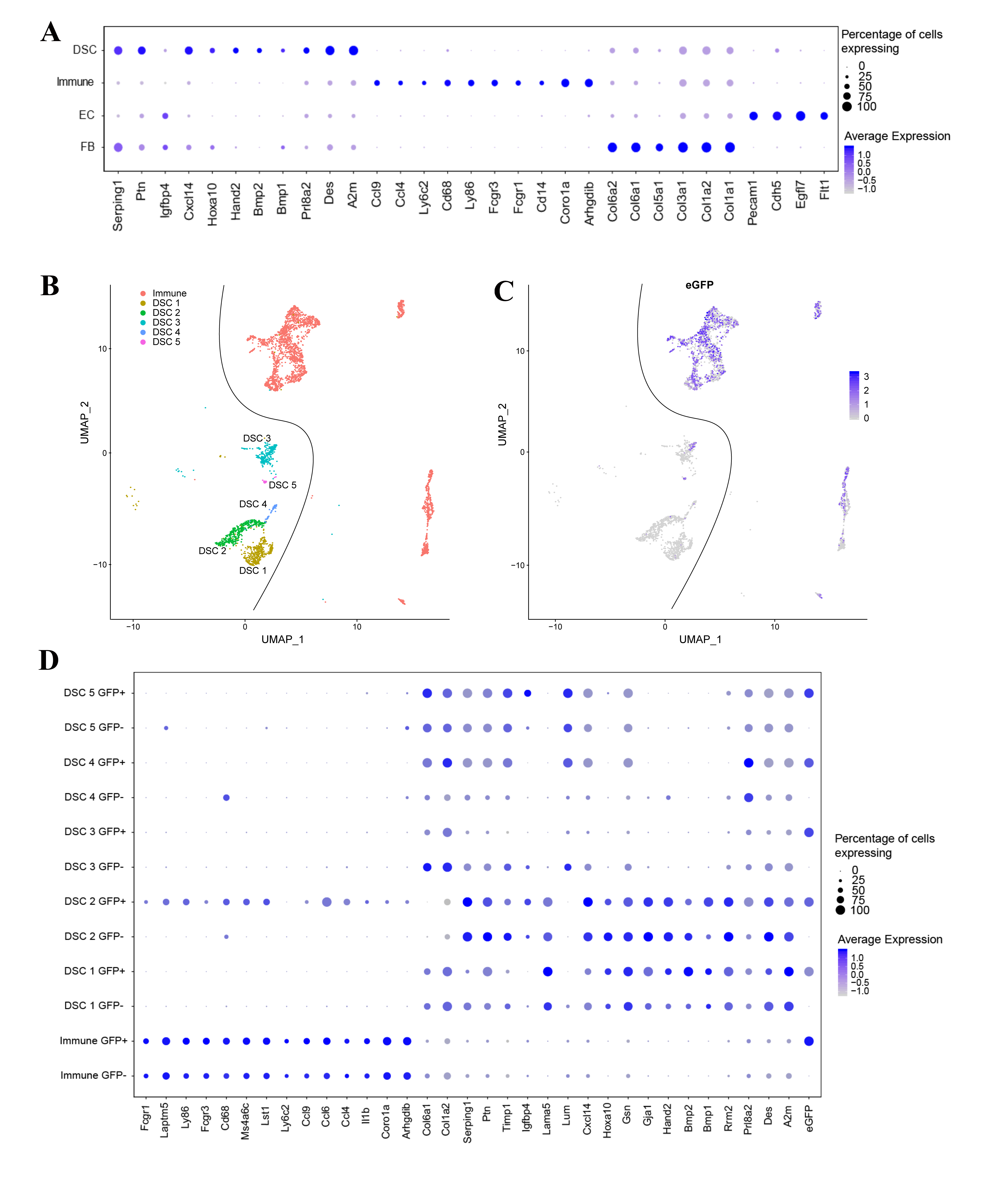

Supplement: S6 Fig — (A) Expression data dot plots of known lineage markers of DSCs, immune cells, ECs, and FBs. (B) Clustering of immune cells and DSCs following UMAP-based visualization of expression differences for cells using established lineage markers and (C) the same plot showing eGFP expression distribution within the same clusters. (D) Expression data dot plots of known lineage markers of eGFP+ and eGFP− cells within immune cell and DSC clusters. DSC, decidual stromal cell; EC, endothelial cell; eGFP, enhanced green fluorescent protein; FB, fibroblast; RNA-seq, RNA sequencing; UMAP, Uniform Manifold Approximation and Projection. (TIF) [file pbio.3000421.s006.tif]

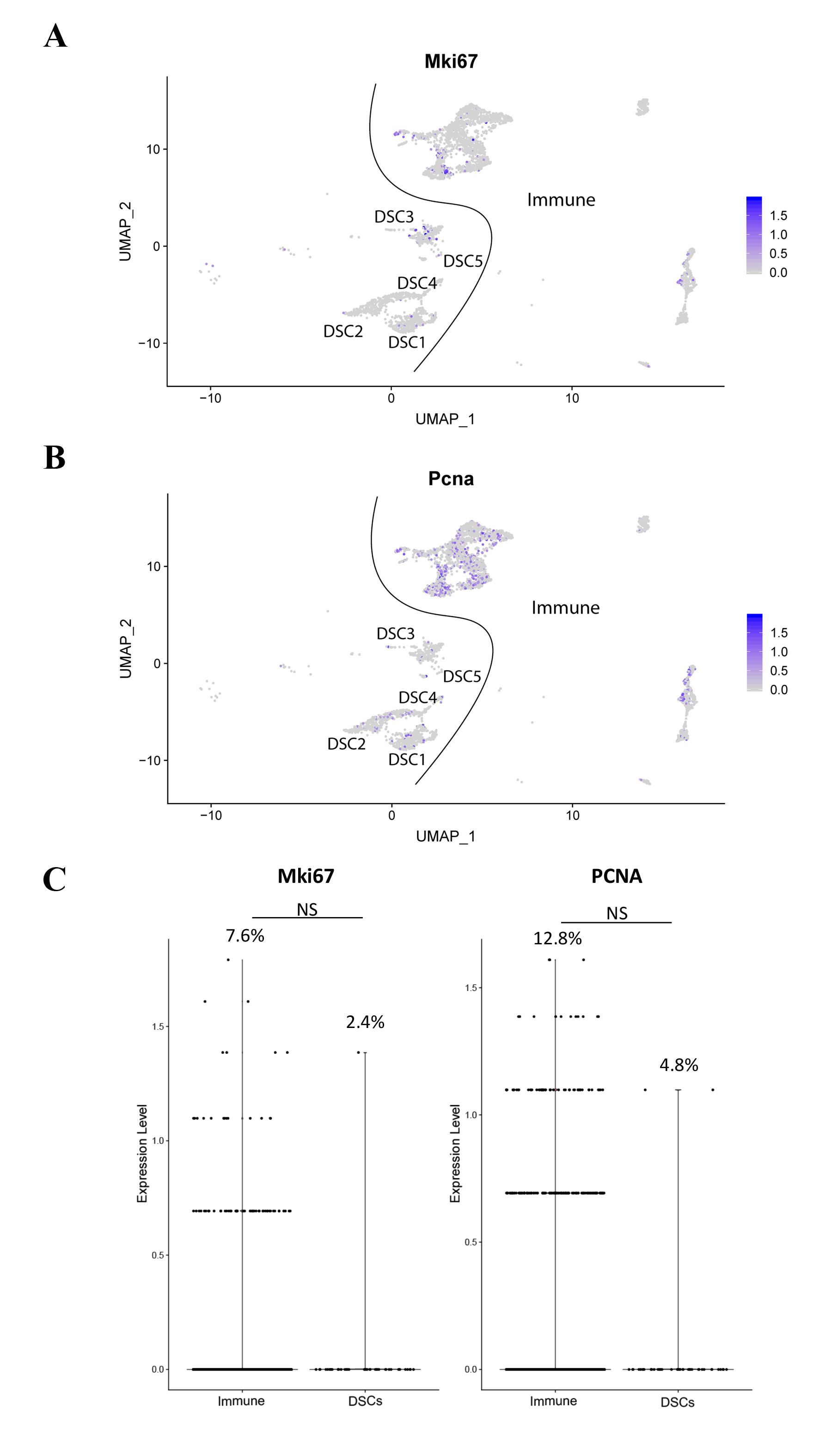

Supplement: S7 Fig — (A and B) Feature plots showing Mki67 (A) and PCNA (B) proliferation marker expression distribution within the total immune cell and DSC clusters. (C) mRNA expression levels and percentage of single Mki67+ and PCNA+ cells within GFP+ immune cells and GFP+ DSCs identified by single-cell RNA-seq. Percentages shown are of cells positive for the respective marker in each group. DSC, decidual stromal cell; GFP, green fluorescent protein; NS, not significant; PCNA, proliferating cell nuclear antigen; RNA-seq, RNA sequencing. (TIF) [file pbio.3000421.s007.tif]

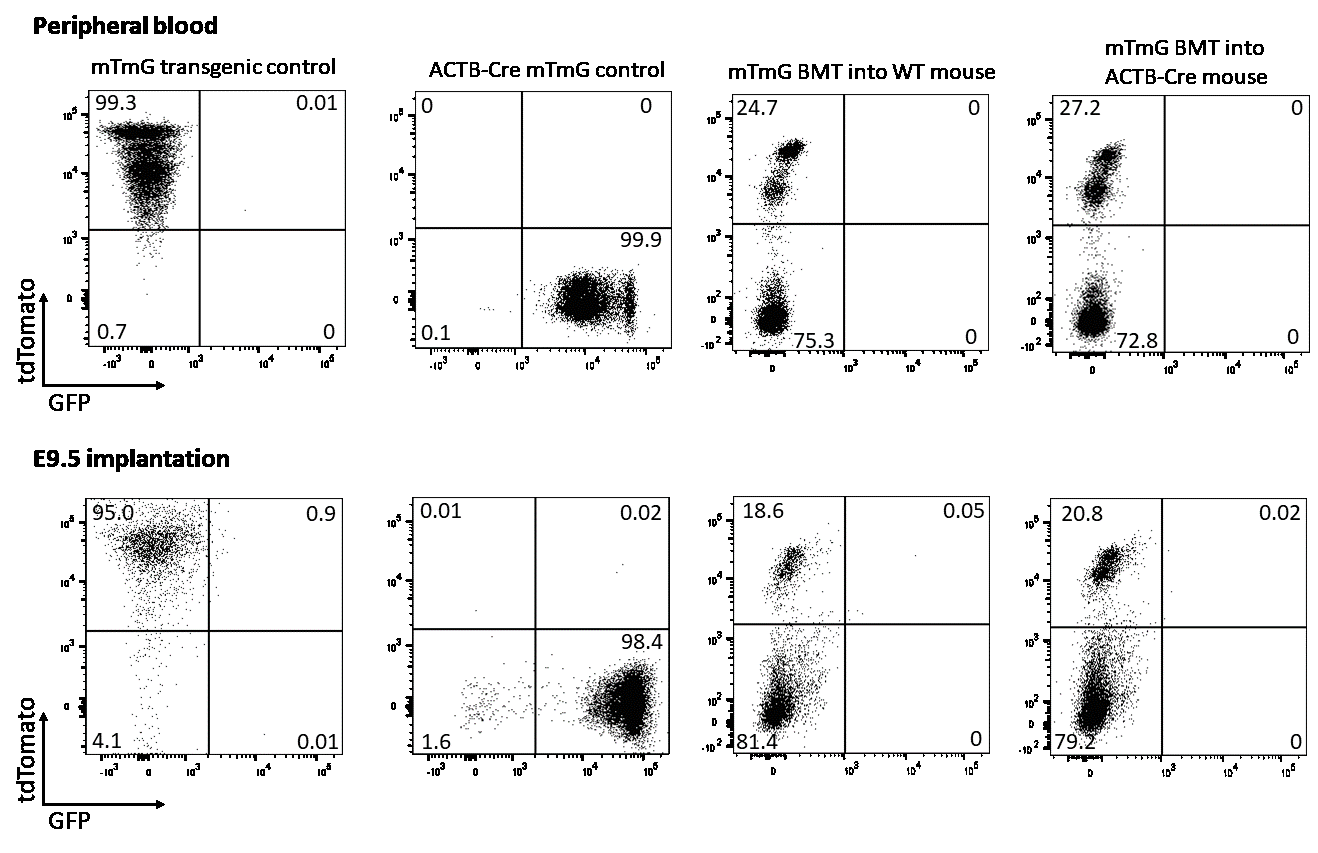

Supplement: S8 Fig — mT/mG transgenic mice were used as BM donors in 5-FU–based BMT into β-actin-Cre (ACTB-Cre) mice expressing Cre ubiquitously. The BM transplants from mT/mG donor into WT mice served as negative controls. Top panel shows flow cytometry of peripheral blood cells from mT/mG transgenic control; mice co-expressing Cre recombinase transgene under β-actin-Cre promoter as positive controls for the efficiency of Cre-mediated conversion from mT to mG in this system; and mTmG BMT into WT (control) or β-actin-Cre mice. Bottom panel shows flow cytometry of dissociated uterine implantation site cells (E9.5) of the same groups. Numbers in each quadrant indicate mean percentage of cells. n = 3–4 mice per group. BM, bone marrow; BMDC, BM-derived cell; BMT, BM transplant; WT, wild-type; 5-FU, 5-fluorouracil. (TIF) [file pbio.3000421.s008.tif]

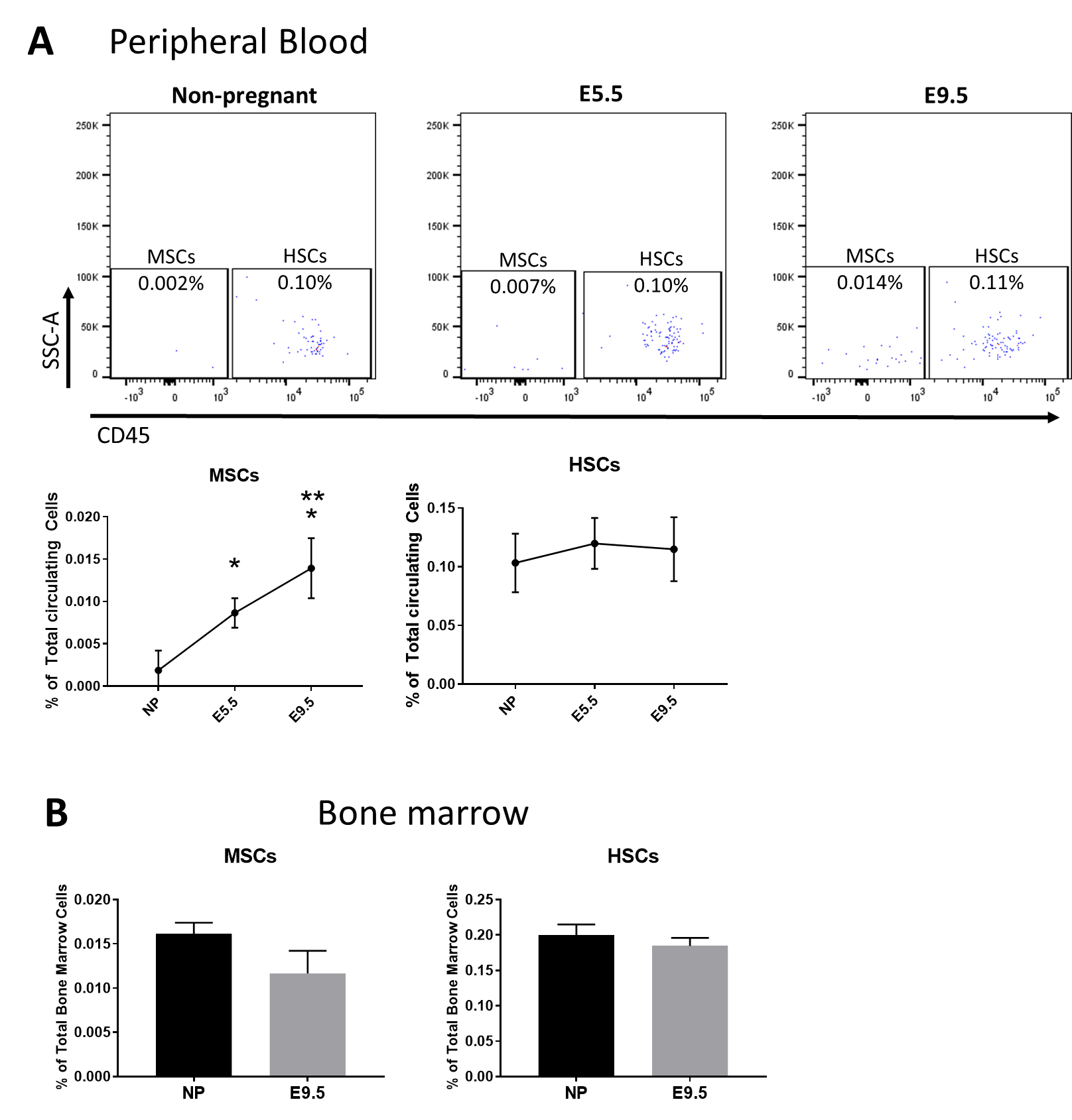

Supplement: S9 Fig — Multicolor flow cytometry was performed on peripheral blood and BM cells of nonpregnant and pregnant mice on E5.5 and E9.5. Cells were gated on Sca1+ and Lin− to identify stem cell populations and further divided into MSCs (Sca1+/CD45−/Lin−) and HSCs (Sca1+/CD45+/Lin−). (A) Representative flow cytometry graphs and quantitation of MSCs and HSCs populations in peripheral blood of nonpregnant, and E5.5 and E9.5 pregnant mice. (B). Quantitation of BM MSCs and HSCs in nonpregnant and E9.5 pregnant mice. n = 3–7 per group. *p < 0.01 versus nonpregnant group. Values and bar graphs represent mean ± SEM. **p < 0.05 versus E5.5 group. Underlying data are available in S1 Data. BM, bone marrow; HSC, hematopoietic stem cell; Lin, lineage; MSC, mesenchymal stem cell; Sca1, stem cell antigen 1. (TIF) [file pbio.3000421.s009.tif]

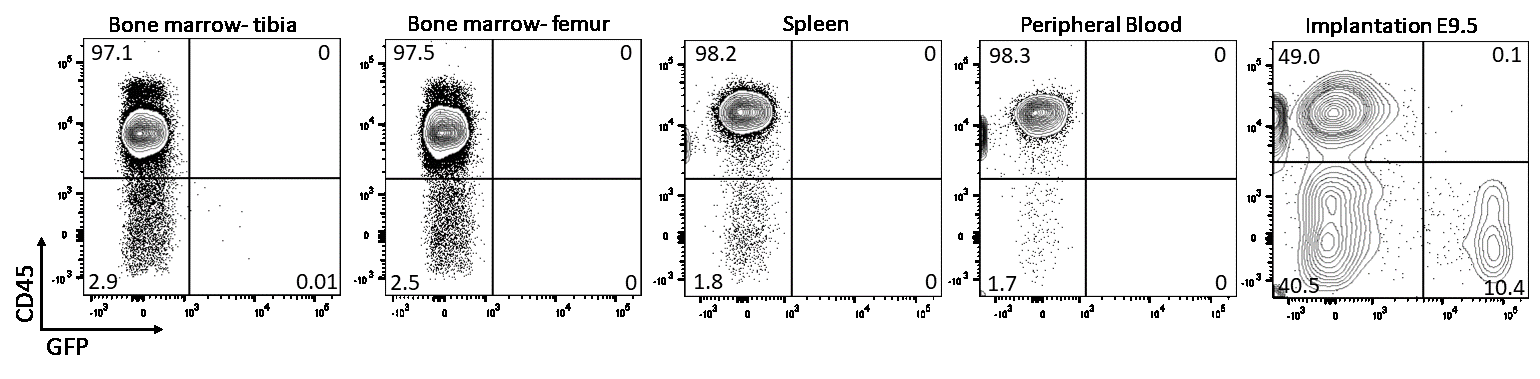

Supplement: S10 Fig — Hoxa11+/− heterozygous mice in which GFP is knocked in, instead of the Hoxa11 gene, were used to identify distribution of Hoxa11 expression. Cells from BM (tibia or femur), spleen, peripheral blood, or implantation site of E9.5 dams were stained with CD45 pan-hematopoietic marker and subjected to flow cytometry analysis (n = 4 mice). BM, bone marrow; GFP, green fluorescent protein; Hoxa11, Homeobox a11. (TIF) [file pbio.3000421.s010.tif]

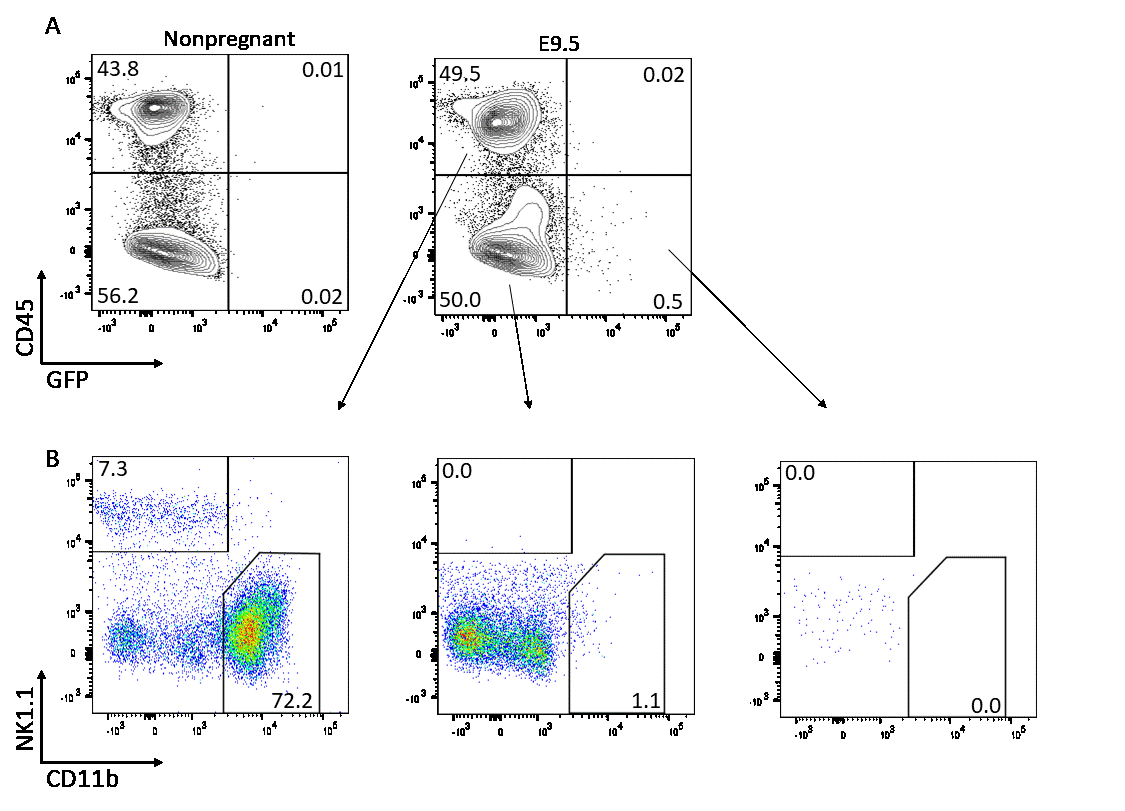

Supplement: S11 Fig — (A) Live single cells of nonpregnant or pregnant (E9.5) uterus showing that BM-derived Hoxa11/GFP+ cells are found in the pregnant uterus and are nonhematopoietic (CD45−). (B) E9.5 uterine cells were gated according to expression of CD45 and Hoxa11-GFP, as indicated by the arrows. NK1.1 and CD11b were used to identify NK cells and myeloid cells, respectively. Numbers in each quadrant indicate mean percentage of cells. N = 3–4 mice per group. BM, bone marrow; GFP, green fluorescent protein; Hoxa11, Homeobox a11; NK, natural killer; WT, wild-type. (TIF) [file pbio.3000421.s011.tif]

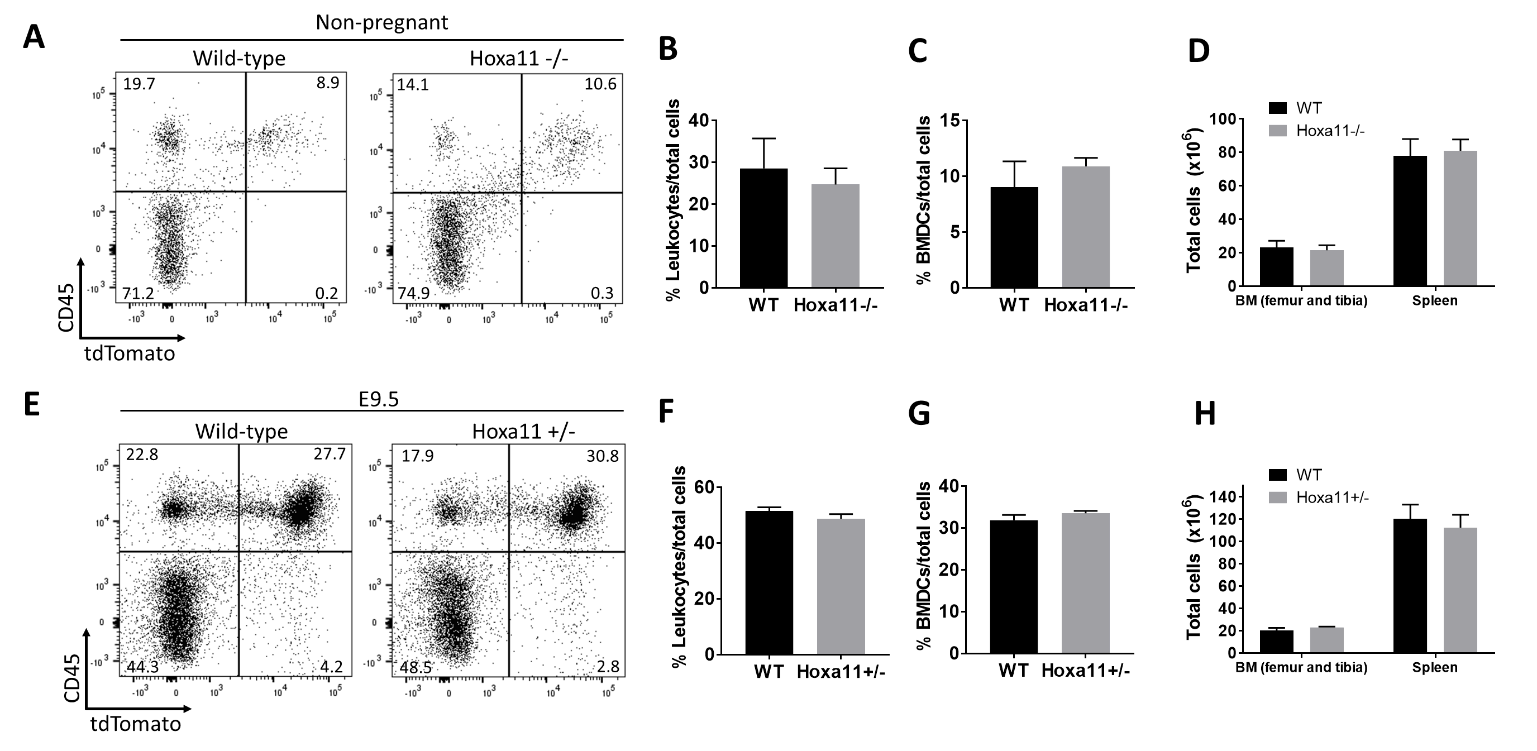

Supplement: S13 Fig — (A-H) Hoxa11−/−, Hoxa11+/−, or WT control mice were transplanted with BM from membrane Tomato (mT) donors. (A and E) Flow cytometry analysis of nonpregnant uterine cells of Hoxa11−/− or WT mice (A) and of E9.5 implantation cells of pregnant Hoxa11+/− and WT mice (E). Cells were stained with CD45 to identify leukocytes, and BMDCs are identified as tdTomato+. Representative graphs are shown. (B, F) Percentage CD45+ leukocytes out of total nonpregnant uterine cells of Hoxa11−/− and WT (B) or out of total pregnant implantation site cells of Hoxa11+/− and WT (F) are shown. (C and G) Percentage tdTomato+ BMDCs out of total nonpregnant uterine cells of Hoxa11−/− and WT (C) or out of total pregnant implantation site cells of Hoxa11+/− and WT (G) are shown. (D and H) Total number of BM cells per one hind limb (femur and tibia) and total number of splenic cells in nonpregnant Hoxa11−/− and WT (D) or pregnant E9.5 Hoxa11+/− and WT mice (H). Bar graphs represent mean ± SEM. n = 3–6 mice per group. Underlying data are available in S1 Data. BM, bone marrow; BMDC, BM-derived cell; Hoxa11, Homeobox a11; WT, wild-type. (TIF) [file pbio.3000421.s013.tif]

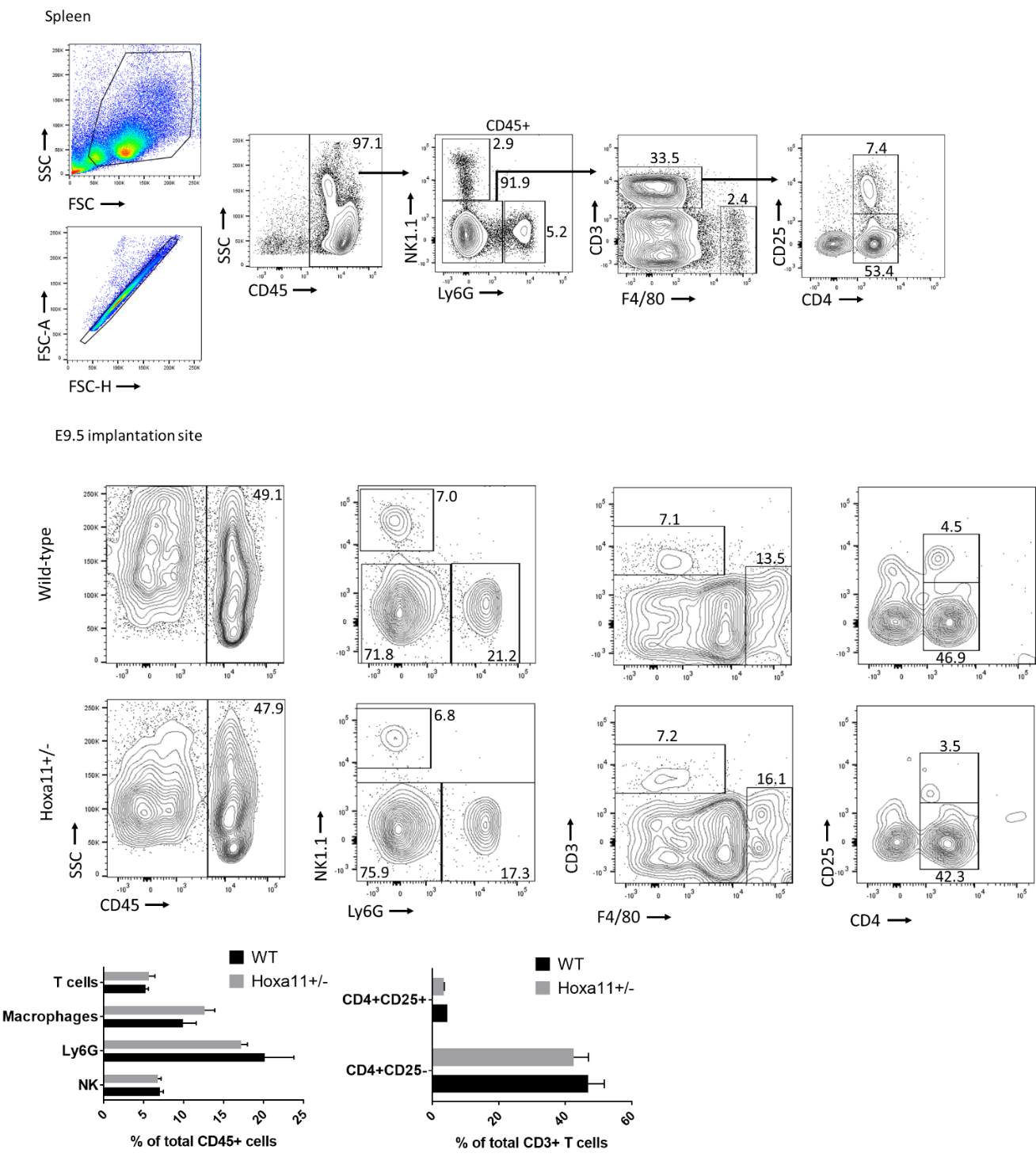

Supplement: S14 Fig — Top panel shows multicolor flow cytometry gating strategy. Live cells were gated according to forward and side scatters to exclude dead cells and debris, followed by single-cell gating with FSC-A and FSC-H. Live leukocyte single cells were gated on CD45. NK1.1 and Ly6G were used to identify NK cells and granulocytes, respectively. CD45+ cells were then gated on NK1.1−Ly6G− to identify T cells (CD3+) and macrophages (F4/80+). T cells (CD3+ NK1.1−Ly6G− F4/80−) were further classified as CD4+CD25+ Treg cells or CD4+CD25− cells. Representative flow cytometry graphs are shown for WT and Hoxa11+/− pregnant mice. Bottom bar graphs represent the mean percentage ± SEM for the various immune subpopulations. n = 4 mice per group. Underlying data are available in S1 Data. FSC-A, forward scatter area; FSC-H, forward scatter height; Hoxa11, Homeobox a11; NK, natural killer; Treg, T regulatory; WT, wild-type. (TIF) [file pbio.3000421.s014.tif]

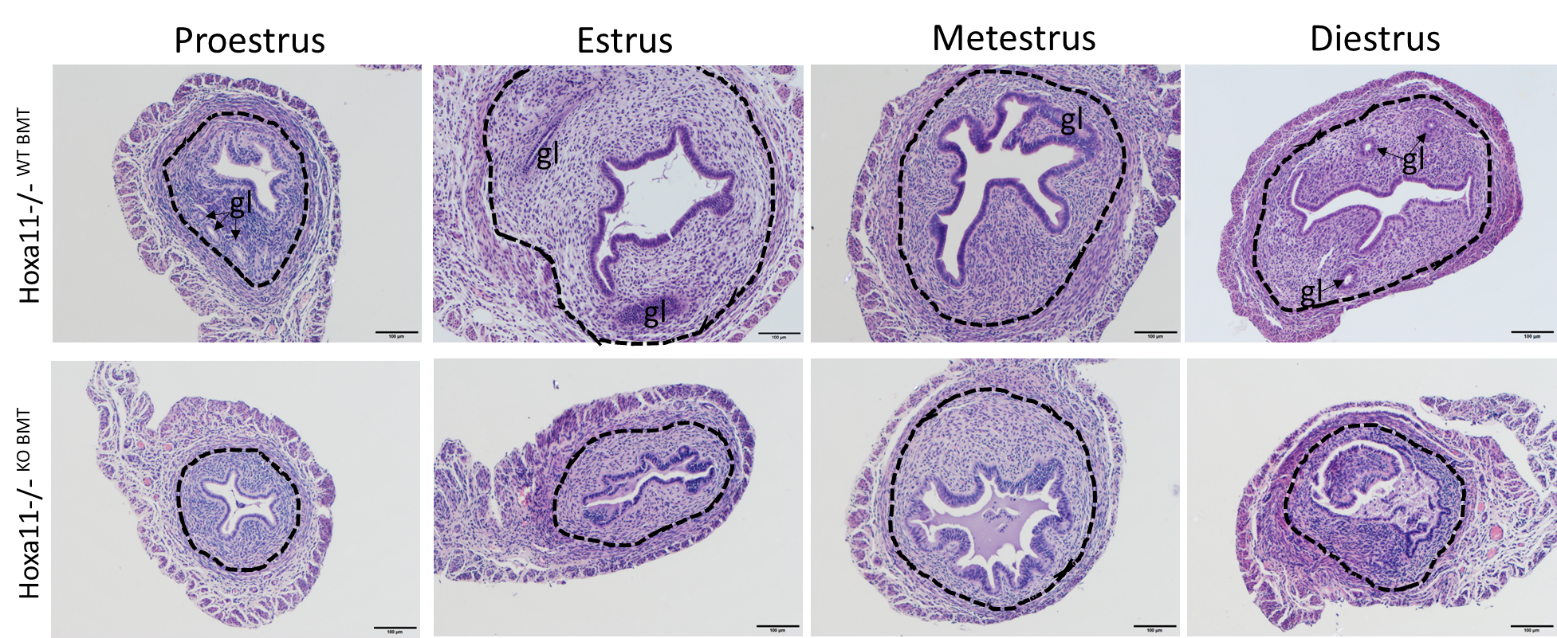

Supplement: S15 Fig — The endometrial area in each photomicrograph is demarcated by a black dashed line. Notice the expanded endometrial stromal area and presence of endometrial glands (gl) only in Hoxa11−/−WT BMT mice. BMT, BM transplant; gl, endometrial gland; HE, hematoxylin–eosin; Hoxa11, Homeobox a11; KO, knockout; WT, wild-type. (TIF) [file pbio.3000421.s015.tif]

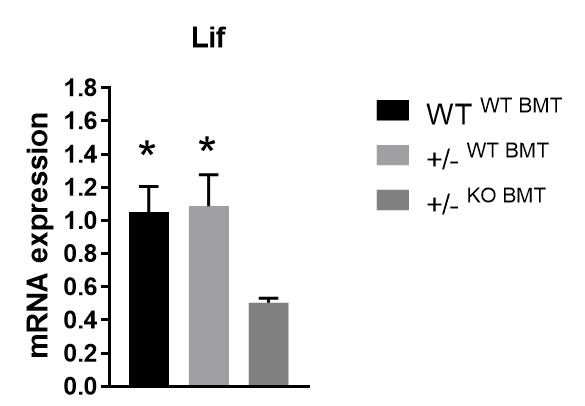

Supplement: S16 Fig — *p < 0.05. Underlying data are available in S1 Data. BMT, BM transplant; Hoxa11, Homeobox a11; KO, knockout; Lif, Leukemia inhibitory factor; WT, wild-type. (TIF) [file pbio.3000421.s016.tif]
