# Supplementary material for: Adult bone marrow progenitors become decidual cells and contribute to embryo implantation and pregnancy
Source: PLoS Biol. 2019 Sep 12;17(9):e3000421. doi: 10.1371/journal.pbio.3000421 (PMC6742226; doi:10.1371/journal.pbio.3000421)
Supplement: S12 Fig — BMT, BM transplant; Hoxa11, Homeobox a11; IPA, Ingenuity Pathway Analysis; KO, knockout; WT, wild-type. (PDF) [file pbio.3000421.s012.pdf]

S12_Fig. Ingenuity Pathway Analysis (IPA) showing significantly enriched pathways of the genes commonly differentially expressed in the comparisons of Hoxa11+/-$^{KO\ BMT}$ vs. Hoxa11+/-$^{WT\ BMT}$, and Hoxa11+/-$^{KO\ BMT}$ vs. WT$^{WT\ BMT}$.

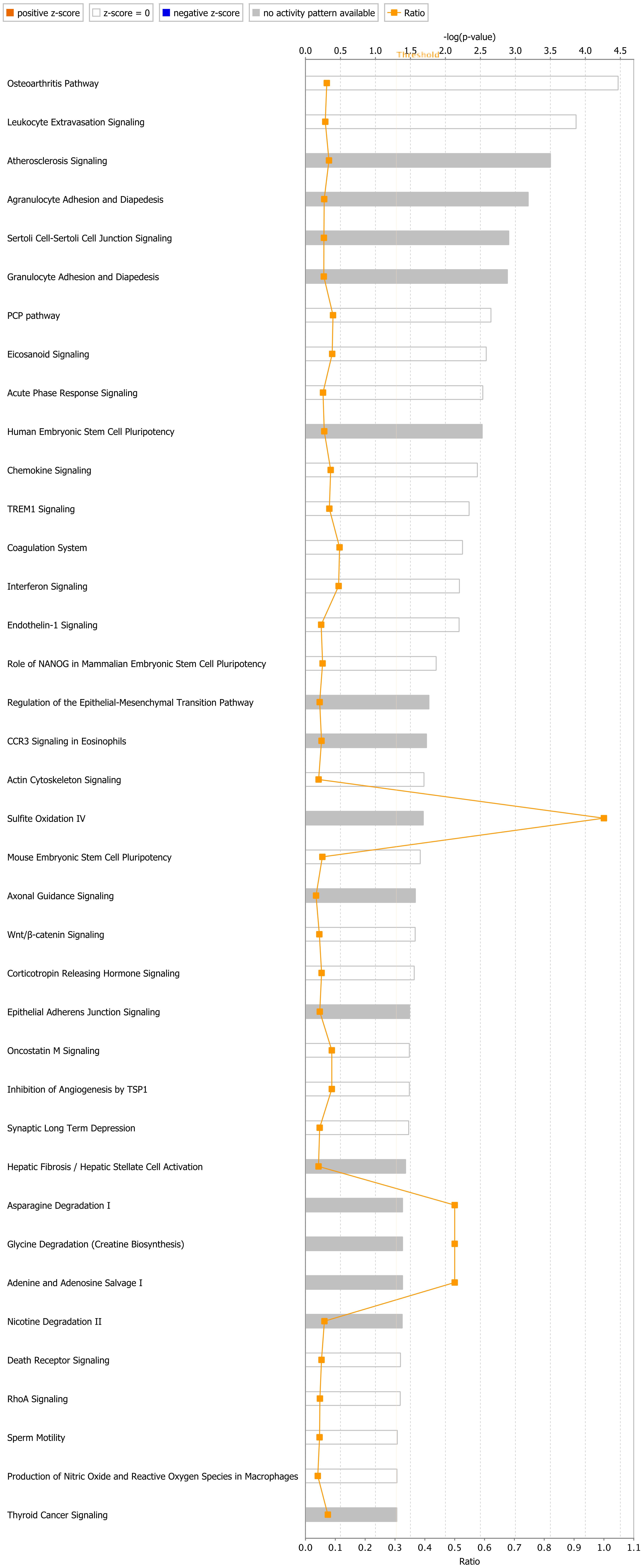

© 2000–2017 QIAGEN. All rights reserved.
