# Supplementary material for: Adult bone marrow progenitors become decidual cells and contribute to embryo implantation and pregnancy
Source: PLoS Biol. 2019 Sep 12;17(9):e3000421. doi: 10.1371/journal.pbio.3000421 (PMC6742226; doi:10.1371/journal.pbio.3000421)
Supplement: S1 Table — (DOCX) [file pbio.3000421.s017.docx]

**S1 Table. Primary and secondary antibodies.**

| Antibody | Manufacturer | Identifier | Concentration |
| --- | --- | --- | --- |
| Goat polyclonal anti-GFP | Abcam | ab6673 | 1:1000 |
| Rabbit polyclonal anti-PCNA | Abcam | ab18197 | 1:4000 |
| PE/Cy7 anti-mouse CD29 | Biolegend | 102221 | 1:200 |
| PE/Cy7 Armenian Hamster IgG Isotype control for CD29 | Biolegend | 400921 | 1:200 |
| PerCp/Cy5.5 anti-mouse CD31 | Biolegend | 102419 | 1:200 |
| PerCP/Cy5.5 Rat IgG2a, k Isotype control for CD31 | Biolegend | 400532 | 1:200 |
| APC anti-mouse CD34 | Biolegend | 119309 | 1:200 |
| APC Rat IgG2a, k Isotype control for CD34 | Biolegend | 400512 | 1:200 |
| PE/Cy5 anti-mouse Sca-1 | Biolegend | 108109 | 1:200 |
| PE/Cy5 Rat IgG2a, k isotype control for Sca-1 | Biolegend | 400509 | 1:200 |
| PE/Cy7 anti-mouse CD44 | Biolegend | 103054 | 1:200 |
| APC/Cy7 Rat IgG2b, k Isotype control for CD44 | Biolegend | 400623 | 1:200 |
| APC/Cy7 anti-mouse CD45 | Biolegend | 103115 | 1:200 |
| APC/Cy7 Rat IgG2b, k Isotype control for CD45 | Biolegend | 400624 | 1:200 |
| PerCp/Cy5.5 anti-mouse CD73 | Biolegend | 127213 | 1:200 |
| PerCP/Cy5.5 IgG1, k Isotype control for CD73 | Biolegend | 400425 | 1:200 |
| PE/Cy7 anti-mouse CD105 | Biolegend | 120409 | 1:200 |
| PE/Cy7 Rat IgG2a, k Isotype control for CD105 | Biolegend | 400521 | 1:200 |
| PerCp anti-mouse CD90.2 | Biolegend | 105321 | 1:200 |
| PerCp Rat IgG2b, k Isotype control for CD90.2 | Biolegend | 400629 | 1:200 |
| PE anti-mouse CD146 | Biolegend | 134703 | 1:200 |
| PE Rat IgG2a, k Isotype control for CD146 | Biolegend | 400507 | 1:200 |
| PerCP/Cy5.5 anti-mouse VEGFR2 | Biolegend | 121917 | 1:200 |
| PerCP/Cy5.5 Rat IgG2a, k Isotype control for VEGFR2 | Biolegend | 400531 | 1:200 |
| APC anti-mouse NK1.1 | Biolegend | 108709 | 1:50 |
| APC Mouse IgG2α, K Isotype control for NK1.1 | Biolegend | 400220 | 1:50 |
| PE anti-mouse CD335 | Biolegend | 137603 | 1:50 |
| PE Rat IgG2a, K Isotype control for CD335 | Biolegend | 400507 | 1:50 |
| APC anti-mouse F4/80 | Biolegend | 123115 | 1:50 |
| APC Rat IgG2α, K Isotype control for F4/80 | Biolegend | 400511 | 1:50 |
| PerCp antimouse Ly-6G | Biolegend | 127654 | 1:50 |
| APC/Cyanine7 anti-mouse/ human CD11b | Biolegend | 101226 | 1:50 |
| Alexa Fluor 700 anti-mouse CD4 | Biolegend | 116022 | 1:50 |
| Brilliant violet 711 anti-mouse F4/80 | Biolegend | 123147 | 1:50 |
| Pacific blue anti-mouse CD3 | Biolegend | 100214 | 1:50 |
| PE anti-mouse CD25 | Biolegend | 101904 | 1:50 |
| Brilliant Violet 605 ^TM^ anti-mouse CD45 | Biolegend | 103140 | 1:50 |
| PE/Cy7 anti-mouse CD11c | Biolegend | 117318 | 1:50 |
| Rat monoclonal anti-CD45 | Abcam | ab25386 | 1:300 |
| Rabbit monoclonal anti-Sca1 | Abcam | ab109211 | 1:200 |
| Rabbit polyclonal anti-HoxA11 | Novus Biologicals | NBP1-83233 | 1:2000 |
| Rabbit polyclonal anti-CD31 | Abcam | ab28364 | 1:100 |
| Rabbit polyclonal anti-PR-H190 | Santa Cruz Biotechnology | sc-7208 | 1:200 |
| Goat polyclonal anti-DPRP | Santa Cruz Biotechnology | sc-379271 | 1:50 |
| Rabbit polyclonal anti-Cytokeratin | Abcam | ab9377 | 1:300 |
| Goat anti-rabbit biotinylated | Vector Laboratories | BA-1000 | 1:200 |
| Donkey anti-goat Alexa Fluor 568 | ThermoFisher Scientific | A11057 | 1:200 |
| Donkey anti-rat Alexa Fluor 488 | ThermoFisher Scientific | A21208 | 1:200 |
| Donkey anti-rabbit Alexa Fluor 488 | ThermoFisher Scientific | A21206 | 1:200 |
| Donkey anti-rabbit Alexa Fluor 568 | ThermoFisher Scientific | A10042 | 1:200 |
| Donkey anti-rabbit Alexa Fluor 647 | ThermoFisher Scientific | A31573 | 1:200 |
