# Supplementary material for: Adult bone marrow progenitors become decidual cells and contribute to embryo implantation and pregnancy
Source: PLoS Biol. 2019 Sep 12;17(9):e3000421. doi: 10.1371/journal.pbio.3000421 (PMC6742226; doi:10.1371/journal.pbio.3000421)
Supplement: S2 Table — (DOCX) [file pbio.3000421.s018.docx]

**S2 Table. Primers for RT-PCR**

| Gene | Primers | Genebank accession number | Product (bp) |
| --- | --- | --- | --- |
| Hoxa11 | F: TTCCGGCCACACTGAGGACAAG  R: ACTCTCGCTCCAGCTCTCGGATCT | NM_010450 | 104 |
| Lif | F: ACGGCAACCTCATGAACCA  R: GGAAACGGCTCCCCTTGA | NM_008501 | 103 |
| Msx1 | F: CAGAGTCCCCGCTTCTCC  R: GTCTTGTGCTTGCGTAGGG | NM_010835 | 74 |
| Wnt4 | F: CATCGAGGAGTGCCAATACCA  R: GGAGGGAGTCCAGTGTGGAA | NM_009523 | 67 |
| Wnt6 | F:CCAGCAGCTCCCTAGGAAAAGT  R: GGCTGGTGTAAACCCCAAGTTC | NM_009526 | 82 |
| Wnt16 | F: CCAAGGAGACAGCATTCATTTATG  R: CTGCATGACCTGGTGACAGAGT | NM_053116 | 70 |
| Fzd6 | F: GCGGCGTTTGCTTCGTT  R: CACAGAGGCAGAAGGACGAAGT | NM_008056 | 67 |
| Foxa2 | F: AGCACCATTACGCCTTCAAC  R: CCTTGAGGTCCATTTTGTGG | NM_010446 | 111 |
| Prl8a2 | F: TACCACAACCCATTCTCAGC  R: GCAGTGATTTCTGGCTCTGA | NM_010088 | 93 |
| Prl6a1 | F: TTTTGAAGTGCAAACTTCTC  R: AGAGTCTGTTTTATTTCTAATG | NM_011166 | 168 |
| Prl3c1 | F: CAAGCACGCACCTGGAAGGGT  R: CCGGTCATATCGTGTGGCAGTCA | [NM_013766](https://www.ncbi.nlm.nih.gov/entrez/viewer.fcgi?db=nucleotide&id=253735808) | 84 |
| Alox12e | F: GCCTGTCATGGTGGCCCT  R: CTTGTCCATGATAGCCAGCTCC | NM_145684 | 106 |
| Alox15 | F: CAGGGATCGGAGTACACGTT  R: GATTGTGCCATCCTTCCAGT | NM_009660 | 186 |
| Jak2 | F: TTGTGGTATTACGCCTGTGTATC  R: ATGCCTGGTTGACTCGTCTAT | NM_008413 | 109 |
| Gapdh | F: GCCTGCTTCACCACCACCTTCTT  R: ATGGCCTTCCGTGTTCCTAC | NM_001289726 | 188 |
